# Efficacies of S-nitrosoglutathione (GSNO) and GSNO reductase inhibitor in SARS-CoV-2 spike protein induced acute lung disease in mice

**DOI:** 10.3389/fphar.2023.1304697

**Published:** 2023-12-08

**Authors:** Judong Kim, Fei Qiao, Avtar K. Singh, Jeseong Won, Inderjit Singh

**Affiliations:** ^1^ Department of Pediatrics, Medical University of South Carolina, Charleston, SC, United States; ^2^ Department of Pathology and Laboratory Medicine, Medical University of South Carolina, Charleston, SC, United States; ^3^ Pathology and Laboratory Medicine Service, Ralph H. Johnson Veterans Administration Medical Center, Charleston, SC, United States; ^4^ Research Service, Ralph H. Johnson Veterans Administration Medical Center, Charleston, SC, United States

**Keywords:** acute lung disease, COVID-19, endotheliopathy, hypercoagulation, inflammation, S-nitrosoglutathione (GSNO), S-nitrosoglutathione reductase (GSNOR), SARS-CoV-2

## Abstract

The severe acute respiratory syndrome coronavirus 2 (SARS-CoV-2), which initially surfaced in late 2019, often triggers severe pulmonary complications, encompassing various disease mechanisms such as intense lung inflammation, vascular dysfunction, and pulmonary embolism. Currently, however, there’s no drug addressing all these mechanisms simultaneously. This study explored the multi-targeting potential of S-nitrosoglutathione (GSNO) and N6022, an inhibitor of GSNO reductase (GSNOR) on markers of inflammatory, vascular, and thrombotic diseases related to COVID-19-induced acute lung disease. For this, acute lung disease was induced in C57BL/6 mice through intranasal administration of recombinant SARS-CoV-2 spike protein S1 domain (SP-S1). The mice exhibited fever, body weight loss, and increased blood levels and lung expression of proinflammatory cytokines (e.g., TNF-α and IL-6) as well as increased vascular inflammation mediated by ICAM-1 and VCAM-1 and lung infiltration by immune cells (e.g., neutrophils, monocytes, and activated cytotoxic and helper T cells). Further, the mice exhibited increased lung hyperpermeability (lung Evans blue extravasation) leading to lung edema development as well as elevated blood coagulation factors (e.g., fibrinogen, thrombin, activated platelets, and von Willebrand factor) and lung fibrin deposition. Similar to the patients with COVID-19, male mice showed more severe disease than female mice, along with higher GSNOR expression in the lungs. Optimization of GSNO by treatment with exogenous GSNO or inhibition of GSNOR by N6022 (or GSNO knockout) protects against SP-S1-induced lung diseases in both genders. These findings provide evidence for the potential efficacies of GSNO and GSNOR inhibitors in addressing the multi-mechanistic nature of SARS-CoV-2 SP-associated acute-lung disease.

## 1 Introduction

Since 2019, more than a million Americans have lost their life due to the coronavirus disease 2019 (COVID-19). COVID-19 is caused by an infection by the SARS-CoV-2 virus. Most patients have mild symptoms, but older individuals (aged 65+) and those with pre-existing comorbidities are more prone to severe complications and a higher risk of morbidity and mortality. Additionally, there is a clear sex-specific bias with males showing a more severe response and higher mortality (Pradhan and Olsson, 2020). The severity and mortality of COVID-19 patients are largely attributed to hypercytokinemia (cytokine storm), vascular dysfunction, and hypercoagulation. However, currently, there are no drugs that can simultaneously address these pathologic events/mechanisms during the acute phase of COVID-19 and post-COVID-19 conditions known as long-haulers. The proposed studies aim to investigate the multi-targeting potential of S-nitrosoglutathione (GSNO) and N6022, an inhibitor of GSNO reductase (GSNOR) for optimizing endogenous GSNO, for targeting SARS-CoV-2 spike protein-induced inflammatory and vascular lung diseases that mimic an acute respiratory disease of COVID-19.

At present, the mechanism underlying SARS-CoV-2-induced acute lung disease is not clear. SARS-CoV-2 is a novel positive-sense single-stranded RNA virus. Therefore, the viral RNA molecules can be recognized by toll-like receptor (TLR) 7 and TLR8 as well as TLR3 when they replicate to double-stranded RNA (Nazmi et al., 2014; Bortolotti et al., 2021) and thereby induce inflammation via activating the NF-κB and IRF-4 signaling pathways (Bortolotti et al., 2021; Manik and Singh, 2022). The SARS-CoV-2 virus infects cells by interaction of the S1 domain of its spike protein (SP) with angiotensin-converting enzyme-related carboxypeptidase-2 (ACE2) present on the surface of host cells (Hoffmann et al., 2020). Recent studies reported that SP binding to ACE2 induces NF-κB activation leading to the expression of proinflammatory cytokines (e.g., TNFα, IL-6, and IL-1β) in human lung cells (Paidi et al., 2021a). SP is also known to induce proinflammatory responses both in mice (C57BL/6) and humans via activating toll-like receptor 4 (TLR4), which mediates Gram-negative bacterial immune responses, or TLR2, which recognizes many bacterial, fungal, and viral substances (Khan et al., 2021; Zhao et al., 2021). It is noteworthy that SP-S1 induces acute lung disease in wild-type mice (C57BL/6) (Paidi et al., 2021a; Paidi et al., 2021b) as well as in transgenic mice expressing human ACE2 (K18-hACE2 mice) (Colunga Biancatelli et al., 2021; Perico et al., 2023). Moreover, recent studies have documented the presence and function of the SARS-CoV-2 SP in the bloodstream of individuals undergoing post-acute sequelae of COVID-19 (Theoharides, 2022; Craddock et al., 2023). These findings underscore the significance of SP in COVID-19-associated acute and long-term disease.

Studies have reported that pulmonary vasculopathy and endotheliopathy are closely linked to Acute Respiratory Distress Syndrome (ARDS) leading to systemic hypoxia in patients with severe COVID-19 (Bonaventura et al., 2021). Patients with severe COVID-19 disease are known to have pulmonary vasoconstriction as well as persistent increases in blood markers of endotheliopathy (e.g., von Willebrand factor/vWF, angiopoietin-2, detached endothelial cells, perivascular immune cell infiltrates) and dysfunctional endothelial barrier (e.g., fibrinogen leakage and endothelial apoptosis) (Goncharov et al., 2017; Jin et al., 2020; Ostergaard, 2021; Martinez-Salazar et al., 2022). Endotheliopathy in COVID-19 disease is caused by multiple mechanisms, including high levels of blood proinflammatory cytokines, recruitment of inflammatory cells, and increased platelet activation, as well as the local increase in angiotensin II (Ang-II) (Kreutz et al., 2020; Shang et al., 2020; Sturrock et al., 2020; Carubbi et al., 2021; Gao et al., 2021). Endotheliopathy frequently accompanies thromboembolism, a severe complication of COVID-19 that is strongly linked to mortality (Merrill et al., 2020; Thachil et al., 2020; Avila et al., 2021). Characteristic changes in hypercoagulation in severe COVID-19 patients include increased fibrinogen and thrombin levels, Factor VIII activity, and circulating vWF as well as exhausted fibrinolysis (Mazzeffi et al., 2021; Rana et al., 2021). The lung is the likely site of macroscopic or microscopic thrombosis in most cases of severe COVID-19 (Srivastava et al., 2020).

Severe COVID-19 patients are known to exhibit lower blood levels of endothelial-derived nitric oxide (NO), increased endothelial oxidative stress and dysfunction, and reduced oxygenation parameters compared to healthy controls (Montiel et al., 2022), thus underscoring the importance of NO availability in severe COVID-19 risk (Nikolaidis et al., 2021). NO is an extra- and intra-cellular signaling molecule regulating diverse physiological processes including immune response, inflammatory response, phagocytic defense mechanism, and cardiovascular homeostasis (Tuteja et al., 2004). NO mediates its physiological actions via activation of soluble guanylyl cyclase (sGC)-dependent cGMP pathway. NO also exerts its physiological actions by reacting with cellular reductant glutathione (GSH) to form S-nitrosoglutathione (GSNO) (Gaston et al., 2003). GSNO exerts its biological activity via post-translational modification of proteins (S-nitrosylation) and is now recognized to regulate various cellular functions related to vascular homeostasis as well as anti-thrombotic, anti-inflammatory, and immunomodulatory processes (Gaston et al., 2003; Kang-Decker et al., 2007; Lowenstein, 2007; Prasad et al., 2007; Haldar and Stamler, 2013; Won et al., 2013). GSNO is more stable than free NO and does not release free NO (Gaston et al., 2003), thus its vascular effect (hypotension) is known to be milder than other conventional free NO donors (de Belder et al., 1994; Broniowska and Hogg, 2012). Cellular GSNO homeostasis is maintained by its synthesis by a reaction between NO (synthesized from nNOS, iNOS, and eNOS) and cellular glutathione (Acevedo et al., 1971), as well as its catabolism, mediated by GSNO reductase (GSNOR). Recently, GSNOR has received increasing attention because of its increased expression/activity under hypoxic and inflammatory conditions and thus loss of tissue GSNO homeostasis causing inflammation and compromised airway function (Marozkina et al., 2015). Currently, several reversible GSNOR selective inhibitors (GSNORi) have been developed, and among them, N6022 has been tested in Phase I and II trials for asthma and cystic fibrosis and proven to meet the safety standard for human use (Colagiovanni et al., 2012).

Aberrant activations of NF-κB, STATs, and IRFs are central cell signaling pathways to hypercytokinemia causing lung inflammation and thrombosis (Matsuyama et al., 2020; Hariharan et al., 2021; Jafarzadeh et al., 2021). Recently, S-nitrosylation is reported to participate in pro-inflammatory cell signaling (Fernando et al., 2019). S-nitrosylation of IKKβ inhibits its activity for IκB phosphorylation and thus NF-κB nuclear transport (Reynaert et al., 2004). Studies from our laboratory and others also reported that S-nitrosylation of NF-κB proteins (p65 and p50) inhibits their interaction with the target gene promoters (Matthews et al., 1996; Marshall and Stamler, 2001; Prasad et al., 2007). Moreover, we also reported that GSNO inhibits IFN-γ-induced STAT1 activation as well as IL-6-induced STAT3 activation (Nath et al., 2010; Won et al., 2013; Kim et al., 2014), and accordingly, GSNO inhibits proinflammatory gene expression (e.g., TNF-α and iNOS) (Khan et al., 2005; Samuvel et al., 2016) as well as immune cell proliferation and tissue infiltration (Kim et al., 2014). We further reported that GSNO/GSNORi modulates the immune balance between effector vs regulatory T helper cells (Treg > T_H_17/T_H_1) and effector vs regulatory functions of B cells by regulating their cytokine expression (IL-10 > IL-6) (Prasad et al., 2007; Nath et al., 2010; Won et al., 2013; Kim et al., 2014; Saxena et al., 2018; Kim et al., 2021b). GSNOR is known to be a key regulator of GSNO in lungs (Que et al., 2009) and GSNORi (N6022) is reported to inhibit NF-κB-mediated pro-inflammatory responses in the lung under inflammatory conditions (Blonder et al., 2014). Therefore, the therapeutic potential of GSNO/GSNORi against SARS-CoV-2-induced lung inflammation is expected to be high.

The anti-platelet efficacy of GSNO has been tested in humans and animals and is reported to reduce the rate of embolization (Molloy et al., 1998; Kaposzta et al., 2001; Kaposzta et al., 2002) at doses that did not cause adverse effects (e.g., hypotension) (de Belder et al., 1994). Mechanistically, GSNO is reported to inhibit platelet activation by inhibiting thrombin-induced cell signaling pathways (Aburima et al., 2017). Our laboratory also reported that GSNO inhibits thrombin-induced endothelial barrier disintegration via inhibiting thrombin-induced intracellular calcium influx and RhoA activation, and subsequent F-actin stress fiber formation (Choi et al., 2019). We also reported that GSNO inhibits matrix metalloprotease (MMP)-mediated degradation of tight-junction proteins and accordingly protects the blood-brain barrier and reduces edema formation in the brain of experimental stroke in rodents (Khan et al., 2012). Moreover, we also reported that GSNO inhibits pro-inflammatory cytokine-induced expression of endothelial cell adhesion molecules (e.g., ICAM-1 and VCAM-1) and thus reduced vascular inflammation and the leukocyte infiltration into the CNS under pathological conditions (Prasad et al., 2007; Won et al., 2013).

Taken together, these studies document the potential of GSNO-mediated mechanisms in SARS-CoV-2-induced inflammation and vascular/endothelial dysfunction. Based on these studies, we evaluated the potential efficacies of GSNO and GSNOR inhibitor (N6022) against SARS-CoV-2 SP-induced acute lung disease in mice.

## 2 Materials and methods

### 2.1 Animal, treatment, and sampling

C57BL/6 J mice were purchased from Jackson Laboratory (Bar Harbor, ME, United States; cat. No. 000664). The GSNOR knockout (GSNOR^−/−^) mice were gifted by Dr. Shyam Biswal of Johns Hopkins University. Mice were supplied with food and water *ad libitum* and kept in ventilated cages in a specific pathogen-free animal care facility maintained by the Medical University of South Carolina throughout the entire study. Animals were housed at a controlled temperature (22°C), humidity (45%–55%), and 12 h light/dark cycle. All animal studies were reviewed and approved by the Medical University of South Carolina’s Institutional Animal Care and Use Committee (IACUC). Eight to nine-week-old male and female C57BL/6 J mice, as well as male GSNOR^−/−^ mice (Casin et al., 2018), were subjected to daily intranasal administration of recombinant extracellular fragment (16–685) of SARS-CoV-2 (2019 nCoV: Accession# 6VSB_A) spike protein S1 subunit (SP-S1) (Mybiosource, San Diego, CA, United States, cat. no. MBS553722) for 10 days. For intranasal treatment, SP-S1 was dissolved in sterilized phosphate-buffered saline (PBS) to achieve a concentration of 100ng/1 μL. Each mouse was given 1 μL (100 ng) in each nostril using a micropipette, totaling 200 ng delivered through both nostrils. Throughout the treatment period, the body temperature and body weight of each mouse were measured daily. Starting from the fifth day of treatment, the administration of SP-S1 was combined with daily treatment of GSNO (1 mg/kg/ip/day; WPI, Sarasota, FL, United States) or N6022 (1 mg/kg/ip/day; Cayman, Ann Arbor, MI, United States). On the 10th day of SP-S1 treatment, the mice were sacrificed for the collection of blood and lung tissues.

### 2.2 Enzyme-linked immunoassay (ELISA)

The concentration of cytokines in the serum was measured by ELISA using the kits for TNF-α (Mybiosource, cat. #: MBS2500421), and IL-6 (Mybiosource, cat. #: MBS2023471). Next, the concentration of blood coagulation factors in the serum was measured by ELISA using the kit for fibrinogen (Abcam, Cambridge, MA, United States, cat. #: ab213478), thrombin (Abcam, cat. #: ab234620), TAT (thrombin anti-thrombin, Abcam, cat. #: ab137994) and vWF (von Willebrand factor, Abcam, cat. #: ab208980).

### 2.3 Quantitative real-time polymerase chain reaction (qPCR)

RNA was extracted from lung tissues using RNeasy protect mini kit (Qiagen, Germantown, MD, United States) based on the manufacturer’s instructions. Total RNA concentration was determined by absorbance at 260 nm using a Nanodrop spectrophotometer (ThermoFisher Scientific, Waltham, MA, United States). cDNA synthesis was performed using iScript cDNA synthesis kit (Bio-Rad, Hercules, CA, United States). For qPCR, the resulting cDNA was mixed with iQ™ SYBR Green Supermix (Bio-Rad) and primer set for TNF-α (Origene, Rockville, MD, United States, cat. #: MP217748), IL-6 (Origene, cat. #: MP206798), IL-10 (Qiagen, cat. #: PPM03017C-200), IFN-γ (Origene, cat. #: MP206683), IL-17 (Origene, cat. #: MP206759), IL-1β (Origene, cat. #: MP206724), or GAPDH (Qiagen, cat. #: PPM02946E-200). The mixture was transferred into a thermal cycler (BIO-RAD CFX96) for PCR amplification.

### 2.4 Immunofluorescence staining

The mice were sacrificed under deep anesthesia and perfused transcardially with phosphate-buffered saline (PBS) and then 4% paraformaldehyde in PBS (pH 7.4). For immunohistochemistry, only the left lung, comprising a single lobe, was utilized. The isolated left lungs were soaked in 4% paraformaldehyde in PBS for 48 h and then in a cryoprotective solution (30% sucrose). The resulting fixed lung tissues were embedded in Scigen Tissue-Plus™ O.C.T. Compound (Thermo Fisher Scientific, Waltham, MA, United States) and then frozen at −80°C. Cryosections (14 μm thick) obtained from the lung were used for immunostaining for GSNOR (Thermo Fisher Scientific, cat. #: 11051-1-AP), CD31 (Thermo Fisher Scientific, cat. #. 14045285), ICAM-1 (Thermo Fisher Scientific, cat. #: MA5407), VCAM-1 (Thermo Fisher Scientific, cat. #: MA5-11447), CD11b (Thermo Fisher Scientific, cat. #: PA5-79532), Ly-6G (Cell Signaling, Danvers, MA, United States, cat. #: 31469s), fibrin (GeneTex, cat. #: GTX19079) and fibrinogen (Abcam, cat. #: ab92572). All digital images were taken using a BX-60 microscope equipped with a DP70 digital camera unit (Olympus, Tokyo, Japan).

### 2.5 Isolation of lung and blood cells for fluorescence flow cytometry

Mice were anesthetized and transcardially perfused with PBS to remove blood cells. Finely minced lung tissues were incubated with 2 mL digestion buffer containing 1 mg/mL collagenase D (Roche, Mannheim, Germany; cat. #: 11,088,866,001) and 50 μg/mL DNAse I (Roche cat. #: 10,104,159,001) in RPMI 1640 medium (Thermo Fisher Scientific cat. #: 61,870,036) at 37°C. The digested tissues were gently mashed onto a 70-μm nylon mesh strainer and then cells were collected by centrifugation (400 x g). The cell pellet was processed using a percoll density gradient centrifugation (Millipore Sigma, cat. #: GE17-0891–02). After centrifugation, the leukocyte-containing layer was carefully collected and subsequently washed with HBSS (Sigma-Aldrich, St. Louis, MO, United States; cat. #: H9269). Then, the resulting cells were cultured in the complete RPMI 1640 media (10% fetal bovine serum, 4 mM L-Glutamine, 200 μg/mL penicillin) containing 50 ng/mL phorbol-12-myristate-13-acetate (PMA), 500 ng/mL ionomycin, BD GolgiPlug™ (brefeldin A; 1 μL per 1 mL media; BD bioscience, San Jose, CA, cat. #: 555,029), and BD GolgiStop™ (monensin; 0.7 μL per 1 mL media; BD bioscience, cat. #: 554,724) for 5 h. The resulting cells were fixed and permeabilized by Fixation/Permeabilization Kit (BD), and stained using specific antibodies, such as anti-CD4-FITC (Biolegend, cat. #: 100,510), anti-CD8-BV-421 (Thermo Fisher Scientific, cat. #: 404–0081-82), anti-IFN-γ-PE (BD bioscience, cat. #. 554,412), anti-TNF-α-PE-Cy7 (Biolegend, cat. #: 506,324), anti-CD11c-BV 605 (Biolegend, cat. #: 117,333), anti-CD45-BV-650 (BD bioscience, cat. #: 103,151), anti-IL-17A-APC-Cy7 (BD bioscience, cat. #: 560,821), and anti-Ly-6G -PerCP-Cy5.5 (Biolegend, cat. #: 127,616). The cells were then washed twice then subjected to flow cytometry using BD LSRFortessa™ Flow Cytometer (BD Bioscience). All flow cytometric data were analyzed using FlowJo software (Treestar, Ashland, OR, United States). For the isolation of blood cells, the blood samples drawn from the mice were subjected to Ficoll density gradient centrifugation (Millipore Sigma, cat. #: F5415). Following the centrifugation, the buffy coat layer including mononuclear cells, platelets, and polymorphic cells was washed with HBSS. Then, the resulting cells were stained for fluorescence flow cytometry using specific antibodies, such as anti-CD31-APC-Cy7 (Biolegend, cat. #: 102,534) for circulating endothelial cells and anti-CD62P-APC (Thermo Fisher Scientific, cat. #: 17–0626-82) for activated platelets.

### 2.6 Evaluation of lung endothelial disruption by evans blue extravasation

Lung endothelial barrier leakage was evaluated using a previously described method (Choi et al., 2019) In brief, the mice were injected intravenously with a 5% solution of Evans Blue (EB) in saline at a dose of 8 mg/kg. The following day, circulating EB in the blood was removed by cardiac perfusion with PBS under deep anesthesia. The lung was then extracted, photographed, and sliced. The lung tissue was homogenized in N, N-dimethylformamide (DMF), followed by centrifugation at 10,000 × *g* for 25 min. The supernatant was analyzed fluorimetrically to measure the content of EB (excitation wavelength: 620 nm, emission wavelength: 680 nm).

### 2.7 Statistical analysis

Statistical analysis and data graphs were performed using GraphPad Prism v8.0 (GraphPad Software, San Diego, CA). One-way ANOVA with Tukey’s multiple comparisons test was used for comparing multiple groups. The bar graph shows the mean ± standard error mean, while the scatter dot plot represents individual data points. For group-level analyses of daily variations in body temperature and body weight, a two-way ANOVA with repeated measures was used. A probability value <0.05 indicates statistical significance.

## 3 Results

### 3.1 Intranasal delivery of SARS-CoV-2 spike protein S1 domain (SP-S1) induces GSNOR expression in the lung of C57BL/6 mice

**FIGURE 1:**
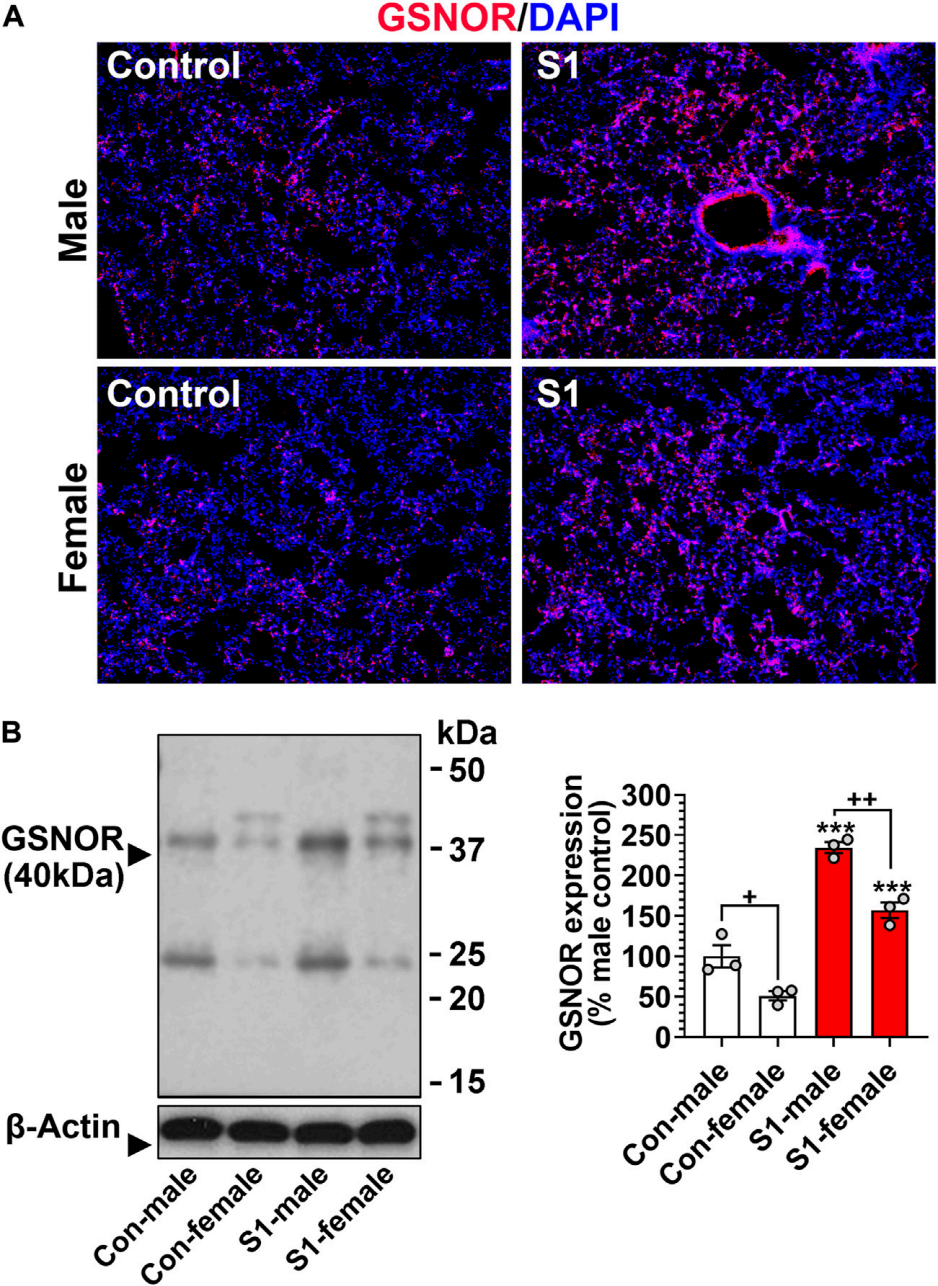
Effects of intranasal SARS-CoV-2 spike protein (S1 domain) delivery on the expression of lung GSNOR in mice. C57BL/6 mice (8–9 weeks old) of both genders were treated with recombinant S1 domain of SARS-CoV-2 spike protein intranasally (100ng/1 μL saline/each nostril) once daily for 10 days. Then, GSNOR expression in the lung was analyzed by immunofluorescence **(A)** and Western analysis **(B)**. Western analysis for β-actin was used for internal loading standard. The bar graph represents the mean ± standard error mean and the scatter dot plot represents an individual data point. ***p ≤ 0.001 vs control mice (each gender) and +++ *p* ≤ 0.001 vs as indicated. *N.S.*: not significant.

Increased GSNOR expression resulting in loss of GSNO homeostasis was reported to cause airway dysfunction in asthma and cystic fibrosis (Marozkina et al., 2015). Therefore, we investigated whether SARS-CoV-2 spike protein induces GSNOR expression in the lungs. Previous studies reported that intranasal delivery of recombinant S1 domain of SARS-CoV-2 spike protein (SP-S1) induces acute lung disease in C57BL/6 mice (Paidi et al., 2021a; Paidi et al., 2021b). We used this model to evaluate the status of GSNOR in the lung in both male and female mice. To assess lung GSNOR expression, male and female C57BL/6 mice were subjected to daily intranasal administration of recombinant SARS-CoV-2 SP-S1 for 10 days. Immunostaining (Figure 1A) and Western analysis (Figure 1B) for GSNOR show that male and female mice had no significant difference in the lung GSNOR protein levels under normal conditions. However, intranasal SP-S1 delivery for 10 days increased the GSNOR levels in both female and male mice, but with greater expression in male mice, suggesting that male mice are likely to have greater dysfunction in lung GSNO homeostasis than female mice in response to intranasal SP-S1 delivery.

### 3.2 Efficacy of GSNO/N6022 against SARS-CoV-2 SP-S1-induced fever and body weight loss

**FIGURE 2:**
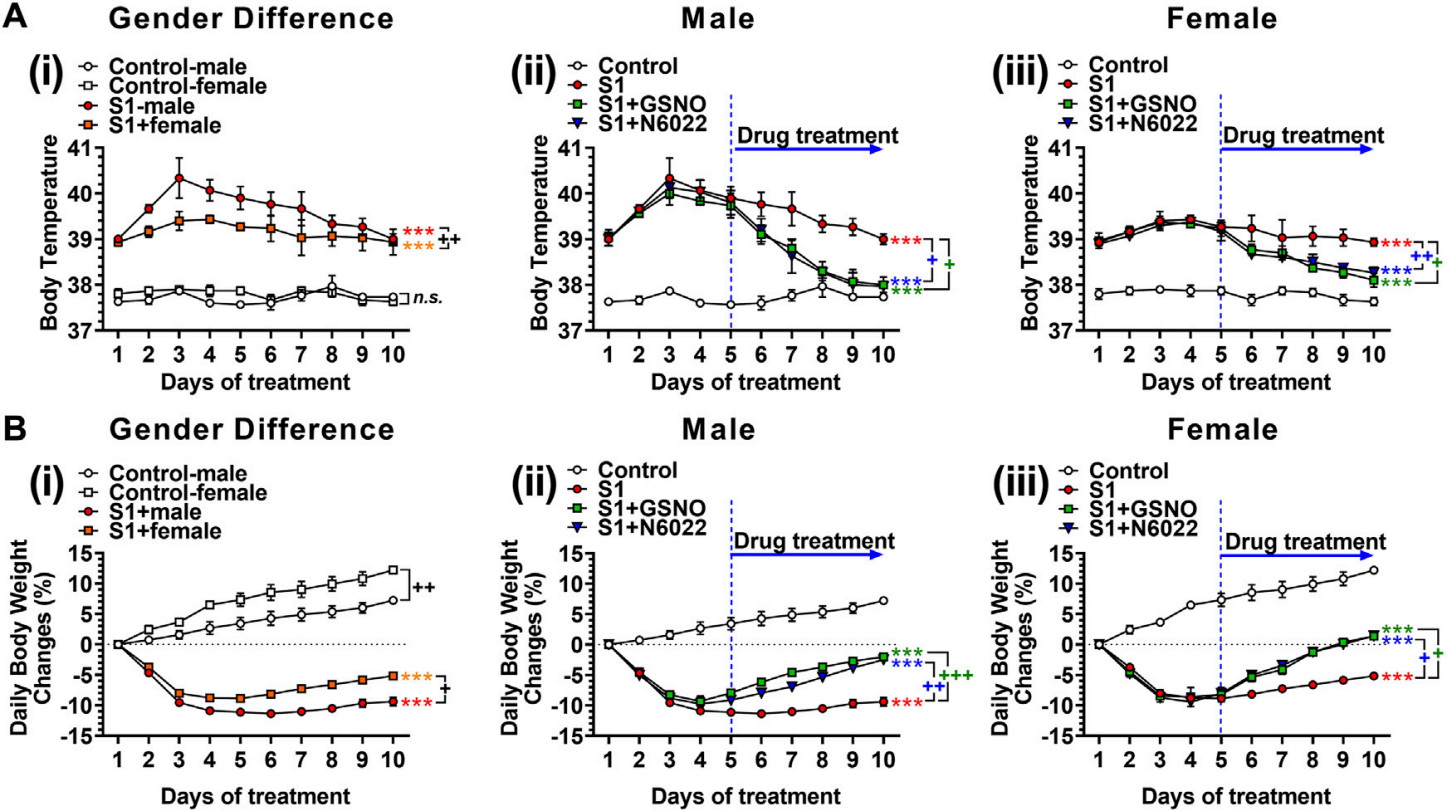
Efficacies of GSNO and N6022 on SARS-CoV-2 spike protein (S1 domain)-induced fever **(A)** and body weight loss **(B)**. Male and female C57BL/6 mice were administered the S1 domain of SARS-CoV-2 spike protein (S1) intranasally on a daily basis for 10 days. **(Ai, Bi)** show differences in body temperature change and body weight between gender. Starting from the fifth day of daily S1 treatment, the mice were additionally treated with GSNO or N6022 (1 mg/kg/ip/day each). Then, the efficacy of GSNO or N6022 on S1-induced increase in body temperature and decrease in body weight in male mice **(Aii, Bii)** and female mice **(Aiii, Biii)** were measured. The line graph represents the mean ± standard error mean. *p < 0.05; **p ≤ 0.01, ***p ≤ 0.001, control vs S1; + *p* ≤ 0.05, ++ *p* ≤ 0.01, +++ *p* ≤ 0.001, as indicated. Please refer to Supplementary Data S1 for an extensive statistical comparison between genders within each treated group.

We next evaluated the efficacy of exogenous GSNO or GSNOR inhibitor (N6022), which optimizes endogenous GSNO, on fever and body weight loss in mice treated with intranasal SP-S1. For this, male and female C57BL/6 J mice were subjected to daily intranasal administration of SARS-CoV-2 SP-S1 for 10 days as described above. COVID-19 has shown sex-specific bias with severe disease and higher mortality among males (Pradhan and Olsson, 2020). Accordingly, we also observed that male mice had a higher fever and greater body weight loss than female mice (Figure 2Ai and Figure 2Bi). Starting from the fifth day of daily SP-S1 administration, the mice were simultaneously treated with GSNO (1 mg/kg/ip/day) or N6022 (1 mg/kg/ip/day) alongside the SP-S1 administration on a daily basis. Both GSNO and N6022 treatments decreased the SP-S1-induced increase in body temperature in males and females (Figures 2Aii, iii). Further, GSNO and N6022 treatments also restored body weight loss significantly in both genders (Figures 2Bii, iii).

### 3.3 Efficacy of GSNO/N6022 against SARS-CoV-2 SP-S1-induced hypercytokinemia (cytokine storm) and lung inflammation

**FIGURE 3:**
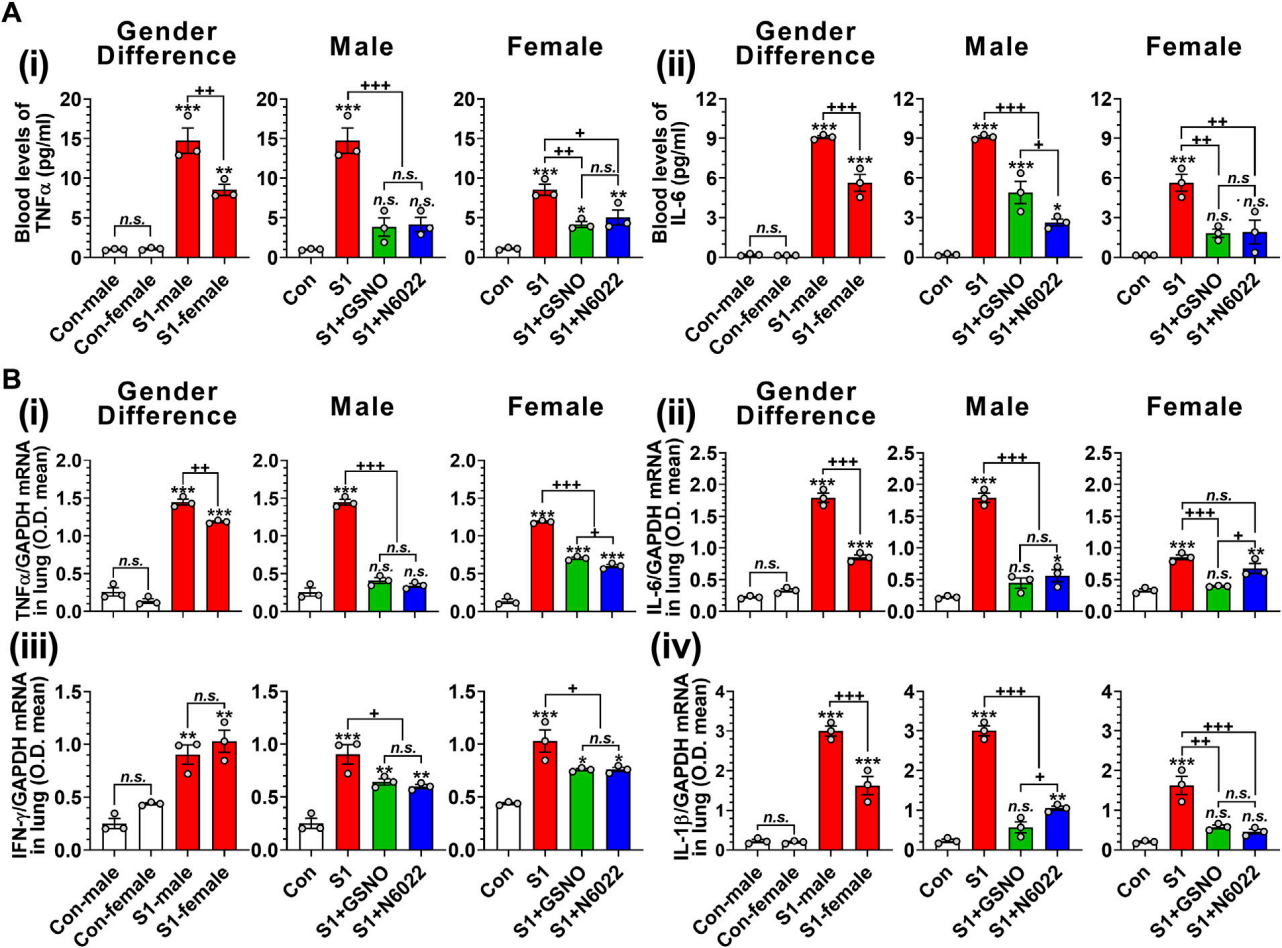
Efficacies of GSNO and N6022 on SARS-CoV-2 spike protein (S1 domain)-induced blood cytokine levels in mice. Male and female C57BL/6 mice were administered the S1 domain of SARS-CoV-2 spike protein (S1) intranasally on a daily basis for 10 days. Starting from the fifth day of daily S1 treatment, the mice were treated with GSNO or N6022 (1 mg/kg/ip/day each). On the 10th day, the mice were euthanized, and the gender-specific differences as well as the efficacy of GSNO or N6022 on blood levels of TNFα **(Ai)** and IL-6 **(Aii)** were analyzed using ELISA. In addition, lung expression of TNFα **(Bi)**, IL-6 **(Bii)**, IFN-γ **(Biii)**, and IL-1β **(Biv)** mRNA was analyzed by quantitative real-time PCR. GAPDH was used for internal control. The bar graph represents the mean ± standard error mean and the scatter dot plot represents an individual data point. *p < 0.05; **p ≤ 0.01, ***p ≤ 0.001 vs control mice and+*p* < 0.05; ^++^ *p* ≤ 0.01, ^+++^ *p* ≤ 0.001 vs as indicated. *N.S.*: not significant. Please refer to Supplementary Data S1 for an extensive statistical comparison between genders within each treated group.

TNFα and IL-6 are reported to play key roles in cytokine storms and are likely to be responsible for the escalation in disease severity of COVID-19 (Liu et al., 2016; Buonaguro et al., 2020; Coomes and Haghbayan, 2020). Therefore, we next measured the blood levels of TNFα and IL-6 on day 10 of intranasal SP-S1 delivery. Figure 3A shows that intranasal SP-S1 delivery increased the blood levels of TNFα and IL-6 in both genders, but more in males, and GSNO/N6022 treatment decreased these increases significantly in both genders (Figures 3Ai, ii). Similarly, intranasal SP-S1 delivery also increased TNF-α, IL-6, IFN-γ, and IL-1β mRNA levels in the lung tissue of both genders and GSNO/N6022 treatments decreased these increases (Figure 3B). Notably, male mice had higher levels of TNF-α, IL-6, and IL-1β mRNA in the lung tissue than female mice but we did not observe any significant differences in IFN-γ mRNA levels between males and females (Figure 3B).

**FIGURE 4:**
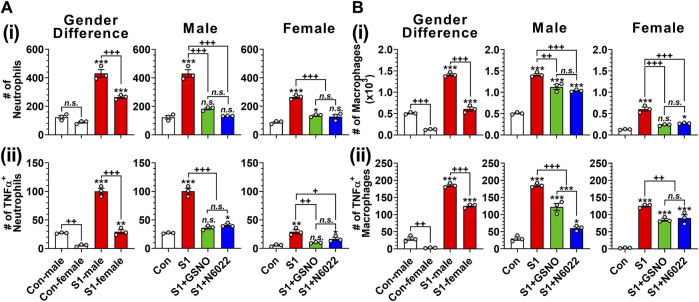
Efficacies of GSNO and N6022 on SARS-CoV-2 spike protein (S1 domain)-induced lung infiltration of neutrophils and macrophages. Male and female C57BL/6 mice were administered the S1 domain of SARS-CoV-2 spike protein (S1) intranasally on a daily basis for 10 days. Starting from the fifth day of daily S1 treatment, the mice were treated with GSNO or N6022 (1 mg/kg/ip/day each). On the 10th day, the mice were euthanized, and the gender-specific differences as well as the efficacy of GSNO or N6022 on lung infiltration of total **(Ai, Bi)** and TNFα^+^ **(Aii, Bii)** neutrophils (CD11cˉ Ly6G^+^) **(A)** and macrophages (CD11c^+^ Ly6Gˉ) **(B)** was analyzed by fluorescence flow cytometry. The bar graph represents the mean ± standard error mean and the scatter dot plot represents an individual data point. *p < 0.05; **p ≤ 0.01, ***p ≤ 0.001 vs control mice and+*p* < 0.05; ^++^ *p* ≤ 0.01, ^+++^ *p* ≤ 0.001 vs as indicated. *N.S.*: not significant. Please refer to Supplementary Data S1 for an extensive statistical comparison between genders within each treated group.

Infiltration and hyperactivation of neutrophils and macrophages are known to be critical for lung inflammation and the development of severe ARDS following the SARS-CoV-2 infection (Hazeldine and Lord, 2021; Knoll et al., 2021). Therefore, we investigated the number of CD11c^+^ Ly6G^−^ cells including alveolar macrophages, and CD11c^−^ Ly6G^+^ cells including lung neutrophils (Misharin et al., 2013). Figure 4Ai and Figure 4Bi show that intranasal SP delivery increased the lung infiltration of neutrophils and macrophages in both genders but males had a greater infiltration than females. In both genders, both GSNO and N6022 treatments decreased the lung infiltration of neutrophils and macrophages (Figure 4Ai and Figure 4Bi). Activated neutrophils and alveolar macrophages are known to release TNF-α in response to acute lung injury (Bhatia et al., 2012). We also observed that intranasal SP-S1 delivery increased the number of TNF-α^+^ neutrophils and macrophages in the lungs of male and female mice but with greater numbers in male mice. GSNO/N6022 treatments decreased the TNF-α^+^ neutrophils and macrophages in both genders (Figure 4Aii and Figure Bii).

**FIGURE 5:**
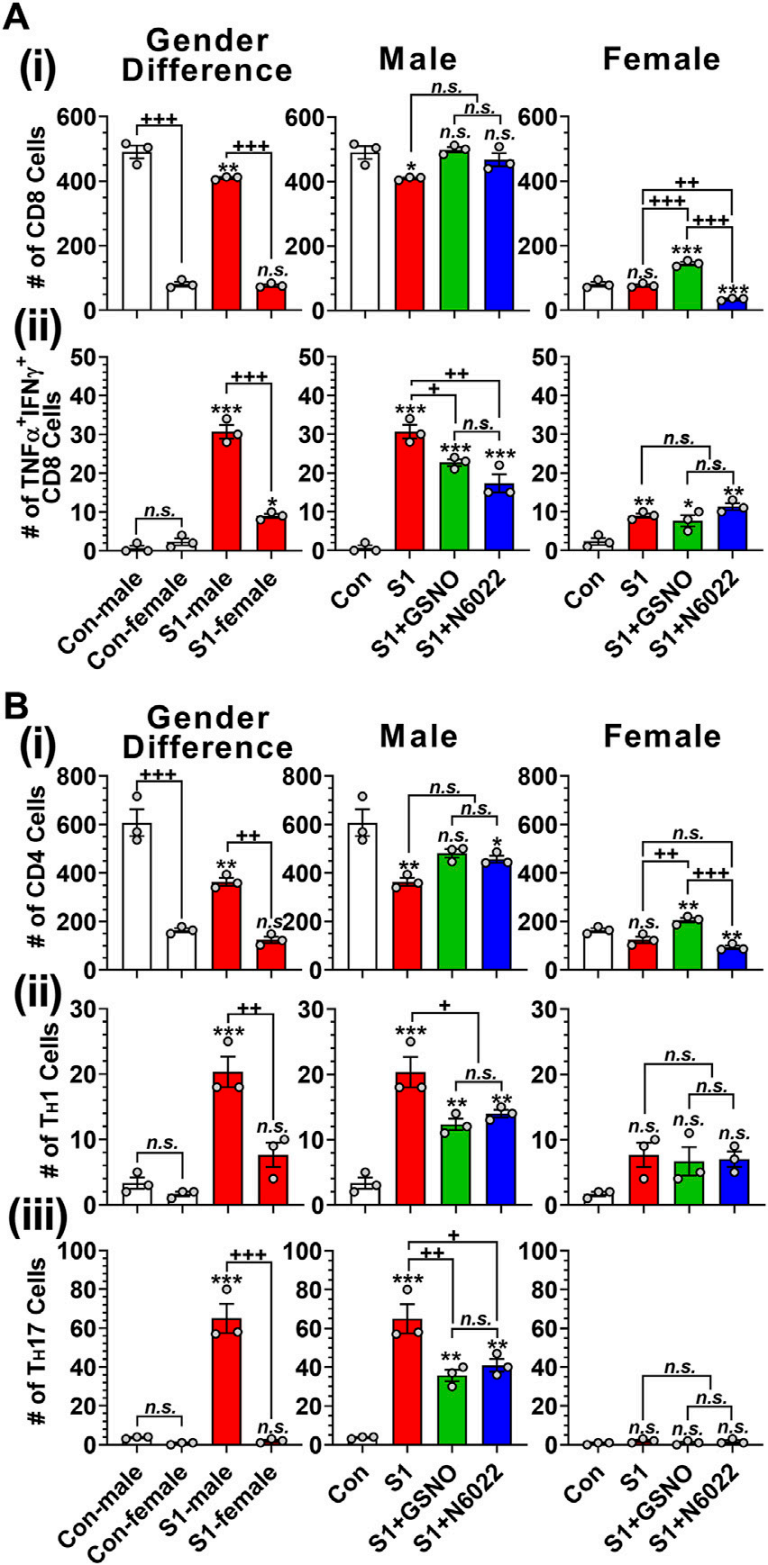
Efficacies of GSNO and N6022 on SARS-CoV-2 SP (S1 domain)-induced lung infiltration of T cells. Male and female C57BL/6 mice were administered the S1 domain of SARS-CoV-2 spike protein (S1) intranasally on a daily basis for 10 days. Starting from the fifth day of daily S1 treatment, the mice were treated with GSNO or N6022 (1 mg/kg/ip/day each). On the 10th day, the mice were euthanized, and the gender-specific differences as well as the efficacy of GSNO or N6022 on lung infiltration of total **(Ai)** and TNFα^+^ IFNγ^+^ **(Aii)** cytotoxic T cells (CD3^+^ CD8^+^) was analyzed by fluorescence flow cytometry. In addition, lung infiltration of total **(Bi)**, IFNγ^+^ T_H_1 **(Bii)**, IL-17a^+^ T_H_17 **(Biii)** helper T cells (CD3^+^ CD4^+^) were also analyzed. The bar graph represents the mean ± standard error mean and the scatter dot plot represents an individual data point. *p < 0.05; **p ≤ 0.01, ***p ≤ 0.001 vs control mice and+*p* < 0.05; ^++^ *p* ≤ 0.01, ^+++^ *p* ≤ 0.001 vs as indicated. *N.S.*: not significant. Please refer to Supplementary Data S1 for an extensive statistical comparison between genders within each treated group.

T cells play an important role in antiviral defenses but their over-expression of proinflammatory cytokines is reported to cause adverse outcomes including pulmonary edema and cardiac injury leading to death (Mehra and Ruschitzka, 2020; Xu et al., 2020). Figure 5Ai and Figure 5Bi show that male mice have greater numbers of lung-resident CD8^+^ (cytotoxic) and CD4^+^ (helper) T cells than female mice under normal conditions. Intranasal SP-S1 delivery slightly decreased the numbers of lung CD8^+^ and CD4^+^ cells in females but more in males (Figure 5Ai and Figure 5Bi). Intranasal SP-S1 delivery, however, greatly increased the number of activated CD8^+^ T cells (TNFα^+^ IFNγ^+^ cytotoxic T cell type 1; Tc1; Figure 5Aii) as well as activated effector CD4^+^ T cells (IFNγ^+^ T_H_1 and IL-17a^+^ T_H_17; Figures 5Bii, iii) in males but not in females. It is of interest to note that female mice had a very low number of T_H_17 cells in the lungs as compared to male mice under SP-S1-treated conditions. SP-S1-increased numbers of activated Tc1 and T_H_1/T_H_17 cells were significantly decreased by treatment with GSNO/N6022 (Figure 5Aii, Figures 5Bii, iii). These data document that intranasal SP-S1 delivery-induced lung inflammation and immune responses are greater in males than females and that GSNO/N6022 treatment ameliorates these immune/inflammatory responses in both genders.

### 3.4 Efficacy of GSNO/N6022 against SARS-CoV-2 SP-S1-induced lung vascular pathology

**FIGURE 6:**
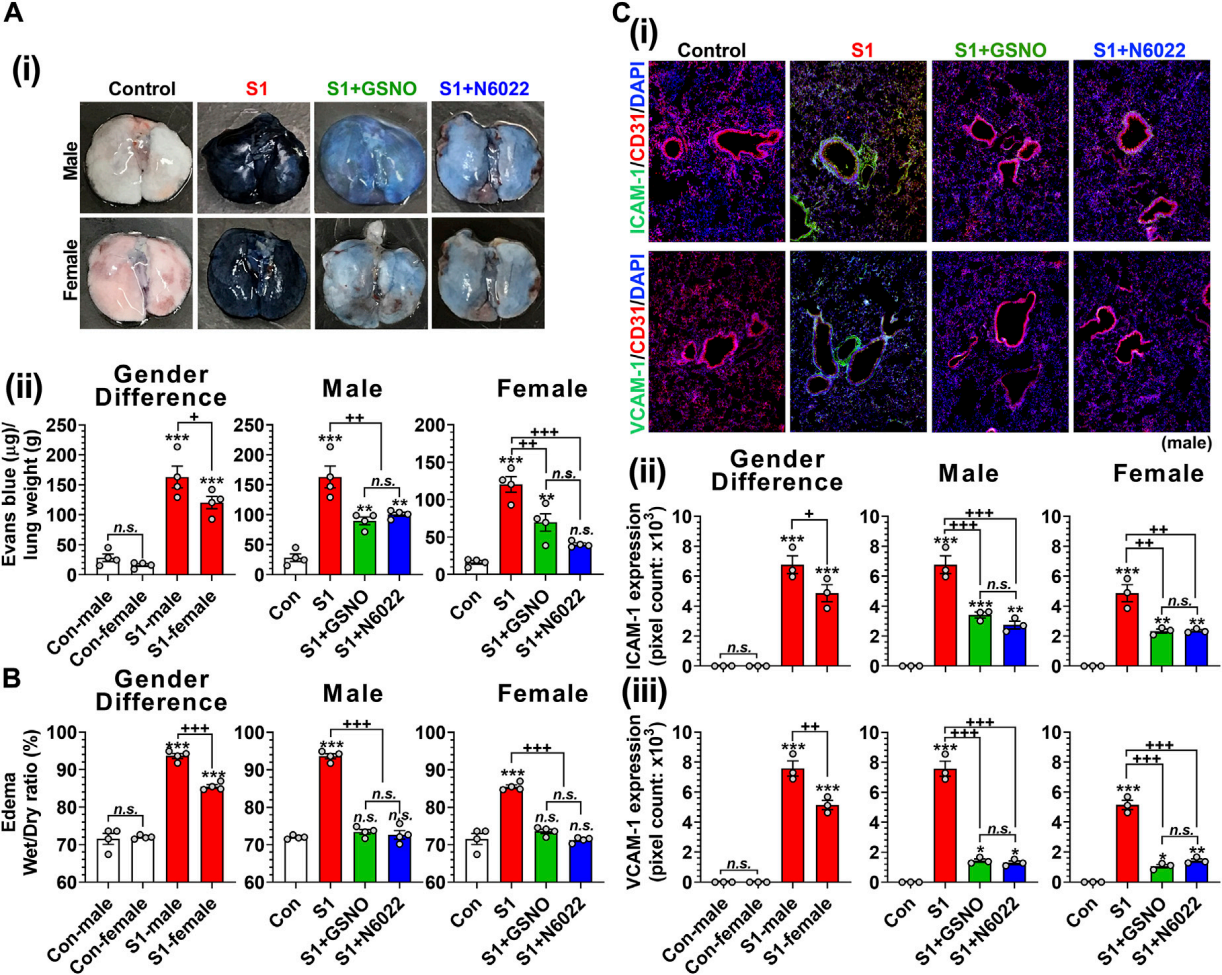
Efficacies of GSNO and N6022 on SARS-CoV-2 SP (S1 domain)-induced lung vascular pathology. Male and female C57BL/6 mice were administered the S1 domain of SARS-CoV-2 spike protein (S1) intranasally on a daily basis for 10 days. Starting from the fifth day of daily S1 treatment, the mice were treated with GSNO or N6022 (1 mg/kg/ip/day each). On the 10th day, the mice were euthanized, and the gender-specific differences as well as the efficacy of GSNO or N6022 on lung vascular hyperpermeability were investigated by Evans blue dye extravasation assay; **(Ai)** shows representative lung photos of male and female mice and **(Aii)** shows quantitative data. In addition, lung edema development (water content) was analyzed by comparing wet vs dry lung weights **(B)**. Lung endothelial (CD31) expressions of ICAM-1 and VCAM-1 were analyzed by immunofluorescence staining of lung sections; **(Ci)** shows representative photos of ICAM-1 and VCAM-1 in male mice and **(Cii, iii)** shows quantitative data (pixel counts). The bar graph represents the mean ± standard error mean and the scatter dot plot represents an individual data point. *p < 0.05; **p ≤ 0.01, ***p ≤ 0.001 vs control mice and+*p* < 0.05; ^++^ *p* ≤ 0.01, ^+++^ *p* ≤ 0.001 vs as indicated. *N.S.*: not significant. Please refer to Supplementary Data S1 for an extensive statistical comparison between genders within each treated group.

Endothelial/epithelial hyperpermeability leading to pulmonary edema formation is recognized as a key pathology of severe COVID-19 (Jin et al., 2020; Zhu et al., 2020; Biering et al., 2021; Bonaventura et al., 2021). To investigate the effect of GSNO and N6022 on intranasal SP-S1 delivery-induced lung endothelial barrier disruption, the mice received Evans blue dye solution intravenously and its extravasation into the lung tissue and the development of lung edema were analyzed. We observed that intranasal SP-S1 delivery increased the extravasation of Evans blue dye from the blood into the lungs (Figure 6A) and induced the development of lung edema (Figure 6B) in both genders but to a greater degree in males and these increases were significantly reduced by GSNO/N6022 therapy (Figures 6A, B).

Patients with severe COVID-19 are known to have increased vascular inflammation characterized by increased expression of lung endothelial ICAM-1 and VCAM-1, which facilitate the infiltration of inflammatory cells into the lung tissue (Birnhuber et al., 2021). Accordingly, we observed that intranasal delivery of mice with SP-S1 resulted in increased expression of ICAM-1 and VCAM-1 in both genders but to a greater degree in males (Figure 6C). We further observed that both GSNO and N6022 treatments decreased the SP-S1-induced increase in ICAM-1 and VCAM-1 expression in the lungs of both genders (Figure 6C).

### 3.5 Efficacy of GSNO/N6022 against SARS-CoV-2 SP-S1-induced thrombotic pathways

**FIGURE 7:**
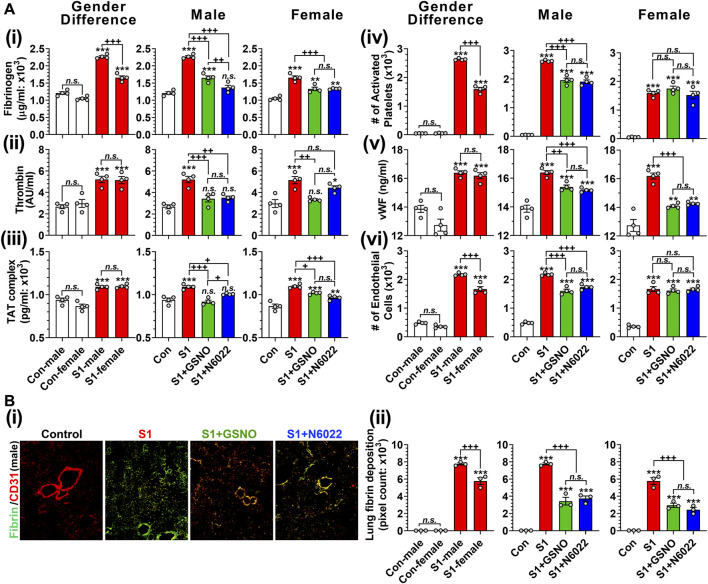
Efficacies of GSNO and N6022 on SARS-CoV-2 SP (S1 domain)-induced thrombotic pathway. Male and female C57BL/6 mice were administered the S1 domain of SARS-CoV-2 spike protein (S1) intranasally on a daily basis for 10 days. Starting from the fifth day of daily S1 treatment, the mice were treated with GSNO or N6022 (1 mg/kg/ip/day each). On the 10th day, the mice were euthanized, and the gender-specific differences, as well as the efficacy of GSNO or N6022 on blood levels of fibrinogen **(Ai)**, thrombin **(Aii)**, thrombin-antithrombin complex **(Aii)**, activated platelets **(Aiv)**, vWF **(Av)**, and circulating endothelial cells **(Avi)**, were analyzed by ELISA and fluorescence flow cytometry. In addition, lung vascular (CD31) deposition of fibrin was also analyzed by immunofluorescence staining of the lung tissue section; **(Bi)** shows a representative photo of male mice and **(Bii)** shows quantitative data (pixel count). The bar graph represents the mean ± standard error mean and the scatter dot plot represents an individual data point. *p < 0.05; **p ≤ 0.01, ***p ≤ 0.001 vs control mice and+*p* < 0.05; ^++^ *p* ≤ 0.01, ^+++^ *p* ≤ 0.001 vs as indicated. *N.S.*: not significant. Please refer to Supplementary Data S1 for an extensive statistical comparison between genders within each treated group.

Pulmonary thrombosis and thromboembolism are the most serious complications among COVID-19 patients (Merrill et al., 2020; Srivastava et al., 2020; Thachil et al., 2020; Avila et al., 2021). The predilection for thrombosis in COVID-19 is driven by at least two distinct, but interrelated, processes: a hypercoagulable state responsible for large-vessel thrombosis and thromboembolism and direct vascular and endothelial injury responsible for *in situ* microvascular thrombosis (Poor, 2021). Hypercoagulation among COVID-19 patients involves increased blood levels of fibrinogen, thrombin, thrombin-anti-thrombin complex, activated platelets, von Willebrand factor (vWF), and circulating endothelial cells (Kichloo et al., 2020; Mazzeffi et al., 2021). Similarly, we observed that mice that received intranasal SP-S1 delivery had increased blood levels of fibrinogen (Figure 7Ai), thrombin (Figure 7Aii), thrombin-antithrombin complex (TAT; Figure 7Aiii), activated platelets (Figure 7Aiv), vWF (Figure 7Av), and circulating endothelial cells detached from endothelium (Figure 7Avi). Notably, we observed male mice had significantly higher blood levels of fibrinogen, TAT, and circulating endothelial cells than female mice in response to the intranasal SP-S1 delivery. In both genders, both GSNO and N6022 treatments decreased the blood levels of those coagulation factors.

Alveolar fibrin deposition is characteristic of diverse forms of acute lung injury (Idell, 2003). Therefore, we next investigated the effect of GSNO and N6022 treatment on the deposition of fibrin in the lung of SP-S1-treated mice. Immunofluorescence staining for fibrin and endothelial cells (CD31) shows that intranasal SP-S1 delivery induces endothelial deposition of fibrin in the lungs (Figure 7Bi). The SP-S1-induced fibrin deposition in the lungs was greater in male mice than female mice and both GSNO and N6022 treatment decreased the fibrin deposition in SP-S1-treated mice (Figure 7Bii).

### 3.6 Effects of GSNOR knockout on intranasal SARS-CoV-2 SP-S1-induced lung disease

**FIGURE 8:**
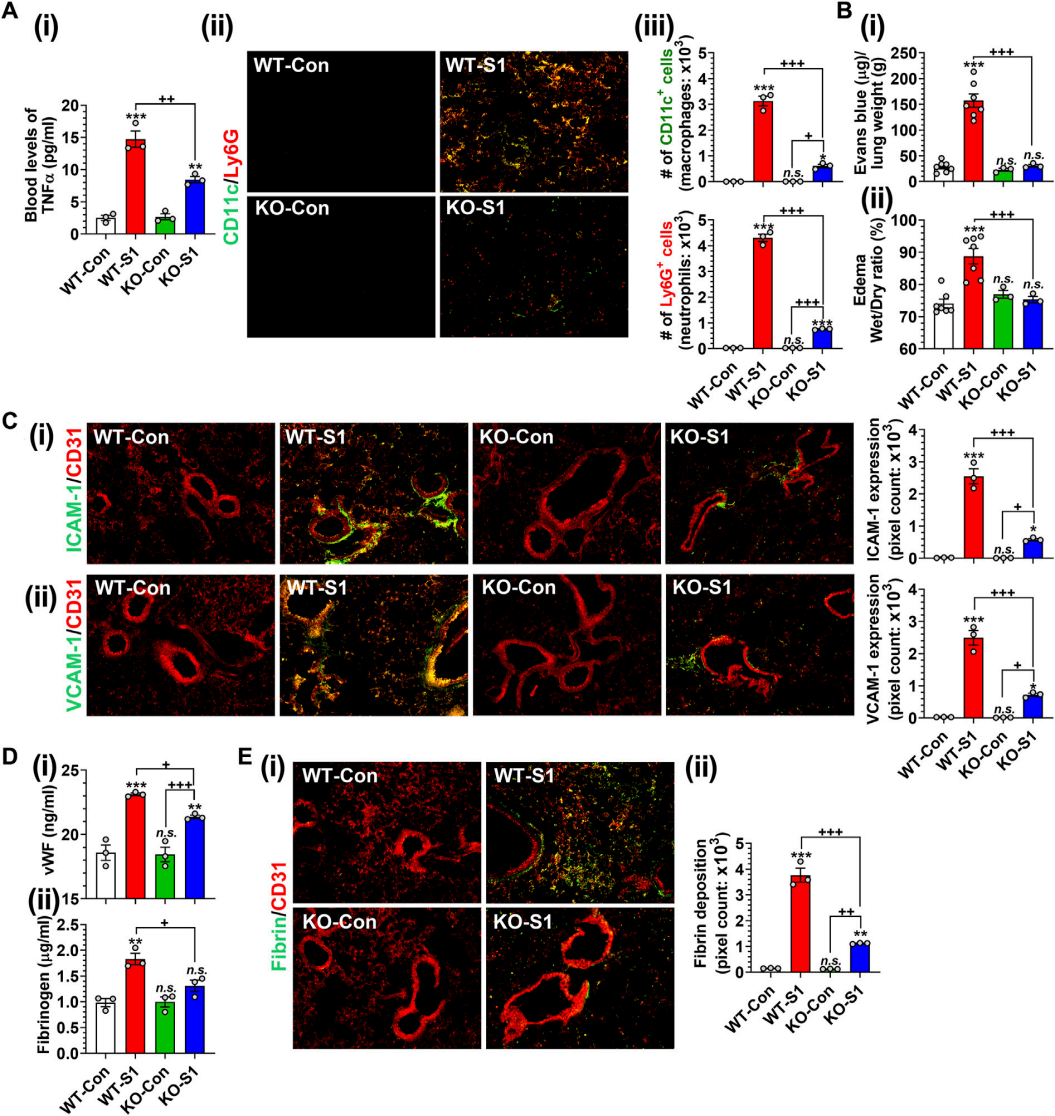
Role of GSNOR in SARS-CoV-2 SP (S1 domain)-induced lung diseases. Male WT (C57BL/6) and GSNOR knockout (KO) mice were administered the S1 domain of SARS-CoV-2 spike protein (S1) intranasally on a daily basis for 10 days and blood levels of TNFα **(Ai)**, lung infiltrations of CD11c+ macrophages and Ly6G + neutrophils **(Aii, iii)**, extravasation of Evans blue into the lungs **(Bi)**, lung edema **(Bii)**, lung vascular (CD31) expression of ICAM-1 **(Ci)** and VCAM-1 **(Cii)**, blood levels of vWF and fibrinogen **(Di, ii)**, lung deposition of fibrin **(Ei, ii)** were analyzed. The bar graph represents the mean ± standard error mean and the scatter dot plot represents an individual data point. *p < 0.05; **p ≤ 0.01, ***p ≤ 0.001 vs control mice and+*p* < 0.05; ^++^ *p* ≤ 0.01, ^+++^ *p* ≤ 0.001 vs as indicated. *N.S.*: not significant.

Next, we investigated the role of GSNOR in SP-S1-induced lung disease using WT (C57BL/6) and GSNOR knockout (KO) mice. We observed that SP-S1-treated GSNOR^−/−^ mice had significantly lower levels of blood TNFα (Figure 8Ai) and lung infiltration of neutrophils (Ly6G^+^) and macrophages (CD11c^+^) (Figures 8Aii, iii) compared to SP-S1-treated WT mice. In addition, GSNOR^−/−^ mice as compared to WT mice showed much lower levels of Evans blue extravasation into the lungs (Figure 8Bi) and lung edema development (Figure 8Bii), lung vascular expression of ICAM-1 and VCAM-1 (Figures 8Ci, ii), blood pro-coagulation factors (vWF and fibrinogen; Figures 8Di, ii), and lung fibrin deposition (Figures 8Ei, ii) in response to intranasal SP-S1 delivery. Collectively, these findings provide compelling evidence for the pathological involvement of GSNOR in SARS-CoV-2 SP-S1-induced acute lung disease in mice.

## 4 Discussion

In this study, we evaluated the efficacies of GSNO and N6022, an inhibitor of GSNOR that optimizes cellular GSNO homeostasis, to alleve SARS-CoV-2 SP-S1-induced acute lung disease in mice. Similar to patients with COVID-19, the mice treated with intranasal SP-S1 showed increased body temperature and body weight loss (Figure 2), increased blood levels and lung mRNA levels of proinflammatory cytokines (Figure 3), lung infiltration by neutrophils, monocytes, and activated cytotoxic and helper T cells (Figures 4, 5) as well as lung vascular hyperpermeability and edema development (Figure 6), and elevation in blood thrombotic factors and lung fibrin deposition (Figure 7). As observed in patients with COVID-19 (Pradhan and Olsson, 2020), SP-S1-induced acute lung disease in mice was more severe in males than in females.

Notably, intranasal SP-S1 delivery also increased the expression of GSNO catabolic enzyme (GSNOR) in the lungs with male mice displaying higher levels than female mice (Figure 1). In addition, the administration of exogenous GSNO, or N6022, for optimizing endogenous GSNO levels confers protection against SP-S1-induced acute lung disease in both male and female mice. Furthermore, GSNOR knockout mice as compared to wild-type mice exhibited reduced inflammatory markers (such as lowered blood levels of TNFα and decreased numbers of infiltrated macrophages and neutrophils in the lungs) in response to intranasal SP-S1 treatment (Figure 8A). GSNOR knockout mice also displayed significantly lower lung vascular pathologies compared to wild-type mice. This was evidenced by decreased lung hyperpermeability (Evans blue extravasation), reduced edema (wet/dry weights ratio), and reduced expression of inflammatory cell adhesion molecules (ICAM-1 and VCAM-1) in mice with SP-S1 treatment (Figures 8B, C). Additionally, GSNOR knockout mice compared to wild-type mice had lower levels of blood coagulation factors (e.g., vWF and fibrinogen) and lung fibrin deposition in response to SP-S1 treatment (Figures 8D, E).

These studies provide compelling evidence that optimizing GSNO through exogenous GSNO treatment or inhibition of GSNOR activity offers protection against SP-S1-induced multi-mechanistic diseases associated with COVID-19, including cytokine storm, hypercoagulopathy, pulmonary embolism, and inflammatory and vascular lung diseases. These findings strongly suggest that GSNOR inhibitor N6022 holds promise as a multi-targeting drug for addressing COVID-19-associated acute lung disease.

While SARS-CoV-2 is reported as not readily infecting conventional laboratory strains of mice due to its low affinity to mouse ACE2 (Lucas et al., 2020), mouse ACE2 does interact with SARS-CoV-2 SP but with less potency than observed with human ACE2 (Ni et al., 2023). Furthermore, SARS-CoV-2 SP-pseudo-typed lentivirus was reported to infect type II alveolar cells in C57BL/6 mice and induce lung inflammation in a receptor binding domain (RBD) dependent manner (Cao et al., 2021). In addition, intranasal treatment of C57BL/6 mice with SARS-CoV-2 SP-S1, identical to SP-S1 used in this study, was also reported to induce acute lung disease in C57BL/6 mice and this effect was inhibited by a specific peptide that inhibits the interaction between SP-S1 and ACE2 (Paidi et al., 2021a; Paidi et al., 2021b; Paidi and Pahan, 2022). SARS-CoV-2 SP-S1 is reported to induce proinflammatory cell signaling via interacting with ACE2 as well as other receptors, such as TLR4 and TLR2 (Khan et al., 2021; Zhao et al., 2021). Although the mechanism underlying SARS-CoV-2 SP-S1-induced acute lung disease is not fully understood, the previously reported studies (Paidi et al., 2021a; Paidi et al., 2021b; Cao et al., 2021; Paidi and Pahan, 2022) and our observations of SP-S1-induced inflammatory and vascular lung pathologies in C57BL/6 mice suggest that this model is useful for understanding of SARS-CoV-2 SP-induced acute lung pathologies.

Glucocorticoids, as broad-spectrum anti-inflammatory agents, primarily exert their function via glucocorticoid receptor-alpha (GRα), mediated by nuclear factor-kB (NF-kB), along with other genomic and non-genomic pathways that ultimately lead to a reduced proinflammatory cytokine expression (Rhen and Cidlowski, 2005). During the pandemic, systemic glucocorticoids were widely used for COVID-19-associated ARDS management as a protective agent against the cytokine storm (Marmor and Jonas, 2020; Salton et al., 2022). However, the therapeutic use of glucocorticoids is associated with many well-known adverse events thus limiting their use. Recently, S-nitrosylation has been recognized to mediate anti-inflammatory cell signaling. S-nitrosylation of IKKβ is reported to inhibit its activity for IκB phosphorylation and thus NF-κB nuclear transport (Reynaert et al., 2004). Studies from our laboratory and others also reported that S-nitrosylation of NF-κB proteins (p65 and p50) inhibits their interaction with the target gene promoters (Matthews et al., 1996; Marshall and Stamler, 2001; Prasad et al., 2007). Moreover, we also reported that GSNO inhibits IFN-γ-induced STAT1 activation as well as IL-6-induced STAT3 activation (Nath et al., 2010; Won et al., 2013; Kim et al., 2014), and accordingly, GSNO inhibits proinflammatory gene expression (e.g., TNF-α and iNOS) (Khan et al., 2005; Samuvel et al., 2016) as well as immune cell proliferation (Kim et al., 2014). GSNOR is known to be a key regulator of GSNO in lungs (Que et al., 2009) and GSNOR inhibitor (N6022) is reported to inhibit NF-κB-mediated pro-inflammatory responses in the lung under inflammatory conditions (Blonder et al., 2014). Similar to these observations, we also observed that the administration of SP-S1-treated mice with GSNO or N6022 decreased the blood levels and lung expression of proinflammatory cytokines (Figure 3). Additionally, GSNO and N6022 treatment also decreased the lung vascular expression of ICAM-1 and VCAM-1 (Figure 6C) and reduced lung infiltration by neutrophils, macrophages, and activated cytotoxic and helper T cells (Figures 4, 5). Moreover, similar observations were also made in GSNOR knockout mice (Figure 8).

Alongside their anti-inflammatory effects, GSNO and N6022 might also alleviate SP-S1-induced acute lung disease by protecting against endothelial dysfunction. Figure 7 shows that GSNO/N6022 treatment decreased the intranasal SP-S1-induced elevations of blood markers related to endotheliopathy (e.g., vWF and circulating endothelial cells). Moreover, the treatments resulted in a decrease in blood coagulation factors induced by SP-S1 treatment, including fibrinogen, thrombin, thrombin-antithrombin complex, and activated platelets, as well as a reduction in lung fibrin deposition (Figure 7). Notably, comparable observations were also made in GSNOR knockout mice (Figure 8).

Endotheliopathy or endothelial dysfunction is a prominent feature of severe COVID-19 including ARDS. Presently, there is no direct evidence indicating that SP-S1 induces lung endotheliopathy leading to ARDS in patients. Nonetheless, numerous animal and *in vitro* studies suggest that SP-S1 may be a direct factor contributing to endotheliopathy under COVID-19-associated ARDS conditions. Notably, endothelial cells express ACE2, functioning as both a SARS-CoV-2 entry mechanism and a signaling receptor (Hamming et al., 2004). Although SARS-CoV-2 typically does not replicate within endothelial cells (Schimmel et al., 2021), its SP has been documented to initiate ACE2-mediated signaling within these cells, leading to the production of proinflammatory cytokines and reactive oxygen species, along with impairment of metabolic function (Kim et al., 2021a; Kumar et al., 2021; Lei et al., 2021; Agostinis et al., 2022). It is noteworthy that the S-nitrosylation of ACE2 inhibits its interaction with SARS-CoV-2 SP (Oh et al., 2023), thus suggesting that GSNO may protect against SP-S1-induced endotheliopathy through S-nitrosylation of endothelial ACE2. GSNO was also reported to inhibit Angiotensin II (Ang II)-dependent Ang II type 1 receptor (AT_1_R)-mediated vasoconstriction of cerebral arteries (Bouressam et al., 2019). SARS-CoV-2 SP binding to ACE2 is reported to increase the local levels of Ang-II by inducing the internalization of ACE2, thus decreasing the cell surface activity of ACE2 for Ang-II conversion to angiotensin (1–7) (Nazerian et al., 2021). Consequently, the increased Ang-II level is known to induce endothelial dysfunction and endotheliopathy (Dandona et al., 2007). Previously, our laboratory reported that GSNO inhibits thrombin-induced endothelial barrier disruption (Choi et al., 2019) and protects against the blood-brain barrier (BBB) disruption under inflammatory and ischemic conditions (Choi et al., 2019). Taken together, these studies indicate that beyond its anti-inflammatory activity, GSNO (or N6022) is likely to protect against lung vascular/endothelial damage by directly acting on endothelial cell signaling.

In humans and animals, GSNO acts as a potent inhibitor for platelet activation and reduces the rate of embolization (de Belder et al., 1994; Molloy et al., 1998). GSNO is also known to inhibit platelet aggregation via S-nitrosylation and inhibition of clotting factor XIII (Catani et al., 1998). Further, GSNO is reported to inhibit fibrinogen polymerization into fibrin by modifying the fibrinogen structure (Chanchikov and Beriia, 1990). These studies in turn suggest GSNO (and N6022) as a stand-alone anti-thrombotic agent against SARS-CoV-2-mediated hypercoagulability and lung thrombosis. Accordingly, we also described that GSNO and N6022 treatment reduces intranasal SP-S1-induced elevations of blood coagulation factors (e.g., fibrinogen, thrombin, thrombin-antithrombin complex, and activated platelets) and lung fibrin deposition (Figure 7). Collectively, these studies propose that the anticoagulation activities of GSNO (N6022) could potentially aid in mitigating the acute lung disease induced by SARS-CoV-2.

COVID-19 exhibits a sex-specific bias, with males experiencing more severe disease and higher mortality rates than females (Pradhan and Olsson, 2020). Similarly, our observations reveal that male mice exhibited a greater extent of acute lung disease compared to female mice in response to intranasal SP-S1 delivery. In addition, male mice express higher levels of GSNOR in the lung than female mice in response to intranasal SP-S1 delivery (Figure 1). At present, it remains uncertain whether the gender-specific bias in GSNOR expression, triggered by SP-S1 treatment, plays a role in the gender-specific severity of acute lung disease. However, the observed mitigation of the acute lung disease by GSNOR inhibitor (N6022) or knockout of GSNOR provides evidence for the pathogenic involvement of GSNOR, suggesting its participation in gender-specific disease severity under SP-S1-induced acute lung disease conditions.

Studies reported that approximately 63% of COVID-19 patients experience T-cell lymphopenia (Lucas et al., 2020). It is of interest to note that even with the decreased counts of CD4^+^ and CD8^+^ T cells, their status was hyperactivated in COVID-19 patients (Xu et al., 2020). Similarly, we observed that intranasal SP-S1 treatment decreased the number of total CD4^+^ and CD8^+^ T cells in the lungs but with increased numbers of TNF-α^+^ IFN-γ^+^ cytotoxic T (Tc) cells and IFN-γ^+^ T_H_1 and IL-17a^+^ T_H_17 cells (Figure 4). These increases were reduced by GSNO and N6022 treatment. Previously, we made similar observations that GSNO and N6022 treatments decreased the number of autoreactive T_H_17 cells in mice with EAE (Nath et al., 2010; Saxena et al., 2018). Interestingly, we observed that male mice had a greater number of Tc, T_H_1, and T_H_17 cells in the lung than female mice (Figures 4B, C). Estrogen has been shown to suppress CD4^+^ and CD8^+^ T cell activations (Khan and Ansar Ahmed, 2015), thus suggesting that the observed sex-specific bias of T cell activation in SP-S1-intoxicated mice may be due to estrogen-related mechanisms. In addition, we also expect that sex-specific bias of lung GSNO expression may also participate in the sex-specific immune/inflammatory responses against the intranasal SP-S1 treatment. At present, the relationship between sex hormone-dependent regulation of GSNOR expression is not understood well, therefore, further studies are necessary.

In recent studies, researchers have highlighted oxidative stress and nitric oxide as pivotal molecular mechanisms underlying both the progression and potential treatments for COVID-19 (Tsermpini et al., 2022). In ARDS patients, dysfunctional eNOS induced by inflammation and oxidative stress plays a critical role in endothelial dysfunction (Guimarães et al., 2021). Moreover, the presence of eNOS polymorphism has been suggested to contribute to the differing mortality rates in COVID-19 between Asian and non-Asian countries (Guan et al., 2020). Notably, levels of nitric oxide were elevated in COVID-19 patients upon hospitalization compared to control groups (Mete et al., 2021). However, severe cases of COVID-19 exhibited lower nitric oxide levels than those with mild or moderate symptoms (Cekerevac et al., 2021). GSNO plays a critical role in nitric oxide signaling in the respiratory track (Que et al., 2009). Therefore, supplementation of exogenous GSNO or an increase in endogenous GSNO by N6022 is a potential therapeutic approach for managing COVID-19 by restoring proper nitric oxide signaling and mitigating endothelial dysfunction caused by oxidative stress and inflammation.

There is a growing body of evidence suggesting that the spike protein contributes to the development of long-COVID syndrome, which is characterized by persistent symptoms following an acute infection with SARS-CoV-2, irrespective of the initial disease’s severity (Theoharides, 2022). Recent studies have shown the presence of spike protein from SARS-CoV-2, along with its viral RNA fragments, in the bloodstream of individuals experiencing post-acute sequelae of COVID-19 for up to 1 year or more after the initial infection (Craddock et al., 2023). In this study, we did not investigate the uptake of SP-S1 by lung tissue, nor its release into the bloodstream in mice subjected to intranasal SP-S1. However, a recent report described that C57BL/6 mice receiving intranasal SP-S1 experienced neuroinflammation and cognitive deficits (Paidi and Pahan). Additionally, we observed that intranasal administration of SP-S1 to C57BL/6 mice led to a dysfunctional blood-brain barrier and increased edema (refer to Supplementary Data S2). At present, it is unclear whether these neurological effects of intranasal SP-S1 arise from its direct release into the bloodstream or from the activation/induction of systemic inflammatory or vascular toxic agents. Nevertheless, exploring the impact of GSNO and GSNOR inhibitors on SARS-CoV-2 SP-mediated long-COVID syndrome in future studies will be of particular interest.

N6022, a specific reversible small molecule GSNOR inhibitor, was developed for the treatment of cystic fibrosis and asthma (Southern et al., 2020). The pharmacokinetics, pharmacodynamics, and toxicology of N6022 have been already studied in humans (Southern et al., 2020). In humans, N6022 treatment up to 40mg/iv/day for 7 days showed no adverse effects and was well-tolerated by healthy volunteers as well as by patients with mild asthma or cystic fibrosis (Southern et al., 2020). In rodents, N6022 treatment up to 10 mg/kg/iv/day was reported to be well tolerated and had no biological adverse effect (Colagiovanni et al., 2012). In the present study, we also did not observe any obvious adverse effect of N6022 (1 mg/kg/ip/day) in SP-S1-treated mice.

In summary, here, we assessed the therapeutic potential of exogenous GSNO and GSNOR inhibitor (N6022) on disease markers observed in human COVID-19 cases using a SARS-CoV-2 SP-S1-induced mouse model of COVID-19-associated acute lung disease. We observed that both GSNO and N6022 treatments decreased the intranasal SP-S1-induced fever and body weight loss as well as elevations of blood levels of proinflammatory cytokines and coagulation factors, lung infiltrations of inflammatory/activated immune cells, and lung fibrin deposition. GSNO is an endogenous compound modulating cell signaling for pro-inflammatory responses, blood coagulation pathways, endothelial permeability, and immune cell modulation. Moreover, recent studies reported that S-nitrosylation of ACE2 inhibits its binding to SARS-CoV-2 SP thus inhibiting/blocking cellular infection of SARS-CoV-2 (Oh et al., 2022). S-nitrosylation is also reported to inhibit palmitoylation of SARS-CoV-2 spike protein and thus inhibits membrane fusion and internalization of the SARS-CoV-2 viral particles (Keyaerts et al., 2004; Akerstrom et al., 2009). Moreover, S-nitrosylation is also reported to inhibit SARS-CoV replication and thus viral RNA production (Akerstrom et al., 2005; Akerstrom et al., 2009). Therefore, GSNO and N6022 seem like promising choices as drugs with multiple targets for treating COVID-19-related acute lung disease. However, further investigation and evaluation are necessary to confirm their effectiveness.

## Data Availability

The original contributions presented in the study are included in the article/Supplementary Material, further inquiries can be directed to the corresponding authors.

## Abbreviations

ACE2, angiotensin-converting enzyme-related carboxypeptidase-2; Ang-II, angiotensin II; ARDS, acute respiratory distress syndrome; COVID-19, coronavirus disease 2019; ELISA, enzyme-linked immunoassay; eNOS, endothelial nitric oxide synthase; GSNO, S-nitrosoglutathione; GSNOR, S-nitrosoglutathione reductase; GSNORi, S-nitrosoglutathione reductase inhibitor; IFN-γ, interferon-γ; IL-1β, interleukin-1β; IL-6, interleukin-6; ICAM-1, intercellular adhesion molecule 1; iNOS, inducible nitric oxide synthase; nNOS, neuronal nitric oxide synthase; NO, nitric oxide; PCR, polymerase chain reaction; SARS-CoV-2, Severe acute respiratory syndrome coronavirus 2; SP-S1, spike protein S1 domain; TAT, thrombin anti-thrombin; Tc1, cytotoxic T cell type 1; TLR, toll-like receptor; TNF-α, tumor necrosis factor-α; VCAM-1, vascular cell adhesion molecule 1; vWF, von willebrand factor.
