# Supplementary material for: Efficacies of S-nitrosoglutathione (GSNO) and GSNO reductase inhibitor in SARS-CoV-2 spike protein induced acute lung disease in mice
Source: Front Pharmacol. 2023 Dec 8;14:1304697. doi: 10.3389/fphar.2023.1304697 (PMC10748393; doi:10.3389/fphar.2023.1304697)
Supplement: Supplementary file 1 [file DataSheet2.PDF]

Supplemental Data 2

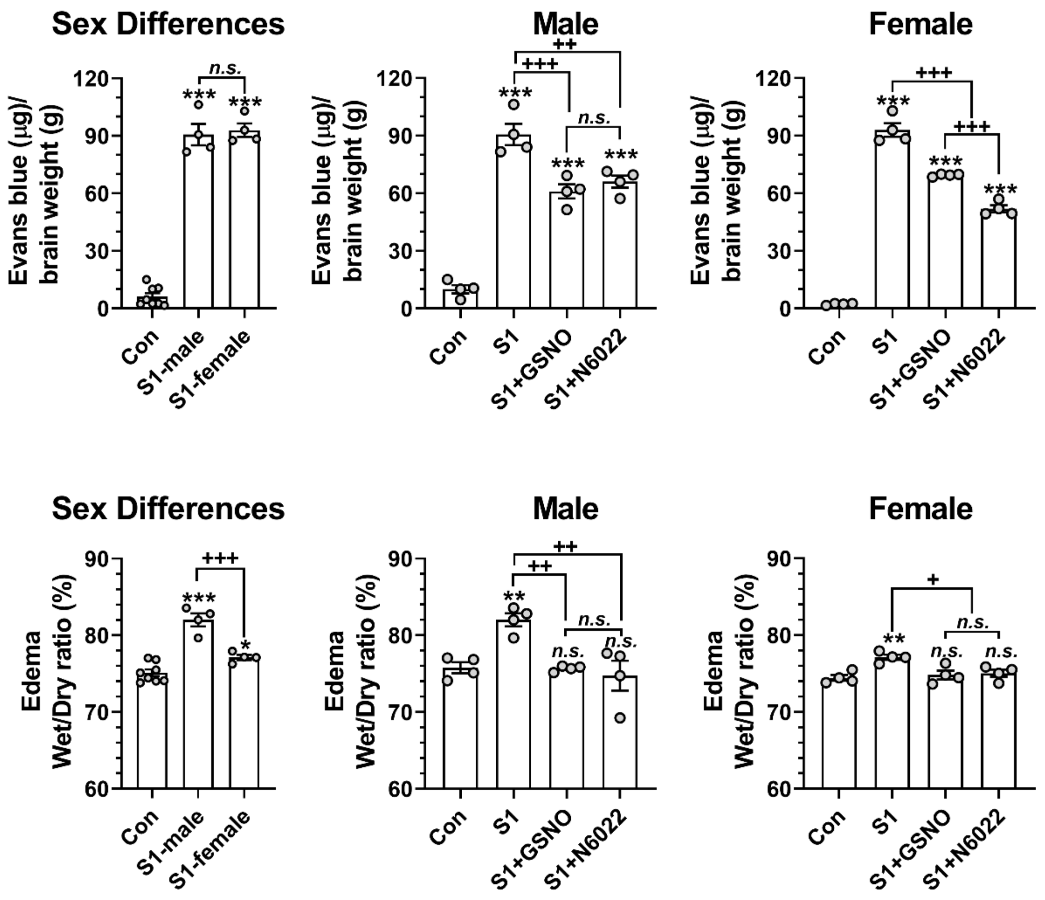

Efficacies of GSNO and N6022 on SARS-CoV-2 SP (S1 domain)-induced brain vascular pathology. Male and female C57BL/6 mice were administered the S1 domain of SARS-CoV-2 spike protein (S1) intranasally on a daily basis for 10 days. Starting from the 5th day of daily S1 treatment, the mice were treated with GSNO or N6022 (1mg/kg/ip/day each). On the 10th day, the mice were euthanized, and the gender-specific differences as well as the efficacy of GSNO or N6022 on brain vascular hyperpermeability were investigated by Evans blue dye extravasation assay. In addition, lung edema development (water content) was analyzed by comparing wet vs. dry lung weights.
