# Supplementary material for: Efficacies of S-nitrosoglutathione (GSNO) and GSNO reductase inhibitor in SARS-CoV-2 spike protein induced acute lung disease in mice
Source: Front Pharmacol. 2023 Dec 8;14:1304697. doi: 10.3389/fphar.2023.1304697 (PMC10748393; doi:10.3389/fphar.2023.1304697)
Supplement: Supplementary file 2 [file DataSheet1.PDF]

Statistical Analysis of figure 2A

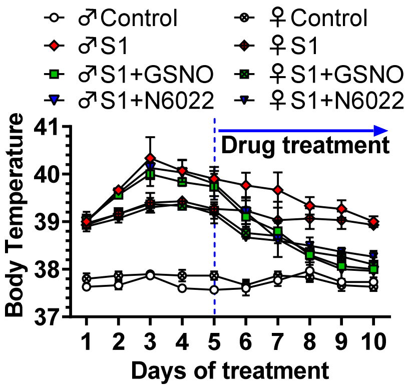

Tukey's multiple comparisons test	Mean Diff.	95.00% CI of diff.	Significant?	Summary	Adjusted P Value
♂ Control vs. ♂ S1	-1.887	-2.234 to -1.539	Yes	****	<0.0001
♂ Control vs. ♂ S1+GSNO	-1.330	-1.800 to -0.8603	Yes	****	<0.0001
♂ Control vs. ♂ S1+N6022	-1.350	-1.869 to -0.8307	Yes	****	<0.0001
♂ Control vs. ♀ Control	-0.08333	-0.2336 to 0.06691	No	ns	0.6585
♂ Control vs. ♀ S1	-1.437	-1.663 to -1.211	Yes	****	<0.0001
♂ Control vs. ♀ S1+GSNO	-1.113	-1.418 to -0.8089	Yes	****	<0.0001
♂ Control vs. ♀ S1+N6022	-1.107	-1.373 to -0.8403	Yes	****	<0.0001
♂ S1 vs. ♂ S1+GSNO	0.5567	0.008320 to 1.105	Yes	*	0.0442
♂ S1 vs. ♂ S1+N6022	0.5367	-0.05351 to 1.127	No	ns	0.0996
♂ S1 vs. ♀ Control	1.803	1.459 to 2.147	Yes	****	<0.0001
♂ S1 vs. ♀ S1	0.4500	0.07263 to 0.8274	Yes	**	0.0096
♂ S1 vs. ♀ S1+GSNO	0.7733	0.3496 to 1.197	Yes	****	<0.0001
♂ S1 vs. ♀ S1+N6022	0.7800	0.3801 to 1.180	Yes	****	<0.0001
♂ S1+GSNO vs. ♂ S1+N6022	-0.02000	-0.6815 to 0.6415	No	ns	>0.9999
♂ S1+GSNO vs. ♀ Control	1.247	0.7793 to 1.714	Yes	****	<0.0001
♂ S1+GSNO vs. ♀ S1	-0.1067	-0.5979 to 0.3845	No	ns	0.9967
♂ S1+GSNO vs. ♀ S1+GSNO	0.2167	-0.3089 to 0.7423	No	ns	0.8925
♂ S1+GSNO vs. ♀ S1+N6022	0.2233	-0.2844 to 0.7311	No	ns	0.8530
♂ S1+N6022 vs. ♀ Control	1.267	0.7495 to 1.784	Yes	****	<0.0001
♂ S1+N6022 vs. ♀ S1	-0.08667	-0.6252 to 0.4519	No	ns	0.9995
♂ S1+N6022 vs. ♀ S1+GSNO	0.2367	-0.3329 to 0.8062	No	ns	0.8867
♂ S1+N6022 vs. ♀ S1+N6022	0.2433	-0.3101 to 0.7967	No	ns	0.8511
♀ Control vs. ♀ S1	-1.353	-1.574 to -1.133	Yes	****	<0.0001
♀ Control vs. ♀ S1+GSNO	-1.030	-1.331 to -0.7295	Yes	****	<0.0001
♀ Control vs. ♀ S1+N6022	-1.023	-1.285 to -0.7615	Yes	****	<0.0001
♀ S1 vs. ♀ S1+GSNO	0.3233	-0.01579 to 0.6625	No	ns	0.0719
♀ S1 vs. ♀ S1+N6022	0.3300	0.02350 to 0.6365	Yes	*	0.0263
♀ S1+GSNO vs. ♀ S1+N6022	0.006667	-0.3581 to 0.3714	No	ns	>0.9999

Statistical Analysis of figure 2B

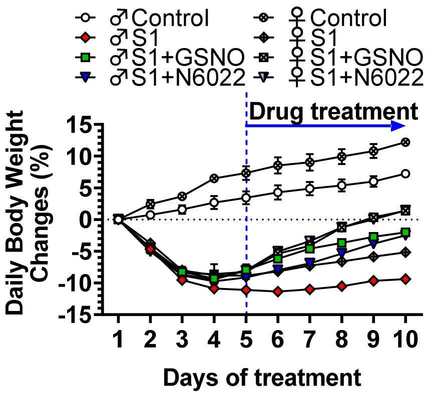

Tukey's multiple comparisons test	Mean Diff.	95.00% CI of diff.	Significant?	Summary	Adjusted P Value
♂ Control vs. ♂ S1	12.44	9.891 to 14.98	Yes	****	<0.0001
♂ Control vs. ♂ S1+GSNO	8.553	6.293 to 10.81	Yes	****	<0.0001
♂ Control vs. ♂ S1+N6022	9.551	7.231 to 11.87	Yes	****	<0.0001
♂ Control vs. ♀ Control	-3.422	-6.176 to -0.6682	Yes	**	0.0059
♂ Control vs. ♀ S1	9.871	7.721 to 12.02	Yes	****	<0.0001
♂ Control vs. ♀ S1+GSNO	7.502	4.810 to 10.19	Yes	****	<0.0001
♂ Control vs. ♀ S1+N6022	7.545	4.874 to 10.22	Yes	****	<0.0001
♂ S1 vs. ♂ S1+GSNO	-3.883	-6.560 to -1.206	Yes	***	0.0007
♂ S1 vs. ♂ S1+N6022	-2.886	-5.612 to -0.1595	Yes	*	0.0308
♂ S1 vs. ♀ Control	-15.86	-18.95 to -12.77	Yes	****	<0.0001
♂ S1 vs. ♀ S1	-2.565	-5.155 to 0.02461	No	ns	0.0539
♂ S1 vs. ♀ S1+GSNO	-4.934	-7.971 to -1.898	Yes	****	<0.0001
♂ S1 vs. ♀ S1+N6022	-4.891	-7.910 to -1.872	Yes	***	0.0001
♂ S1+GSNO vs. ♂ S1+N6022	0.9975	-1.470 to 3.465	No	ns	0.9057
♂ S1+GSNO vs. ♀ Control	-11.98	-14.85 to -9.102	Yes	****	<0.0001
♂ S1+GSNO vs. ♀ S1	1.318	-0.9935 to 3.629	No	ns	0.6271
♂ S1+GSNO vs. ♀ S1+GSNO	-1.051	-3.866 to 1.763	No	ns	0.9350
♂ S1+GSNO vs. ♀ S1+N6022	-1.008	-3.804 to 1.788	No	ns	0.9457
♂ S1+N6022 vs. ♀ Control	-12.97	-15.89 to -10.05	Yes	****	<0.0001
♂ S1+N6022 vs. ♀ S1	0.3202	-2.050 to 2.690	No	ns	0.9999
♂ S1+N6022 vs. ♀ S1+GSNO	-2.049	-4.909 to 0.8117	No	ns	0.3360
♂ S1+N6022 vs. ♀ S1+N6022	-2.006	-4.847 to 0.8363	No	ns	0.3547
♀ Control vs. ♀ S1	13.29	10.50 to 16.09	Yes	****	<0.0001
♀ Control vs. ♀ S1+GSNO	10.92	7.719 to 14.13	Yes	****	<0.0001
♀ Control vs. ♀ S1+N6022	10.97	7.778 to 14.16	Yes	****	<0.0001
♀ S1 vs. ♀ S1+GSNO	-2.369	-5.102 to 0.3640	No	ns	0.1346
♀ S1 vs. ♀ S1+N6022	-2.326	-5.039 to 0.3875	No	ns	0.1437
♀ S1+GSNO vs. ♀ S1+N6022	0.04317	-3.094 to 3.181	No	ns	>0.9999

Statistical Analysis of figure 3A-i

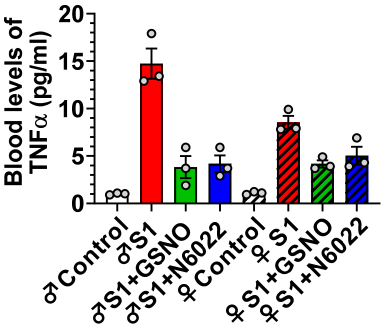

Tukey's multiple comparisons test	Mean Diff.	95.00% CI of diff.	Significant?	Summary	Adjusted P Value
♂ Control vs. ♂ S1	-13.73	-18.03 to -9.430	Yes	****	<0.0001
♂ Control vs. ♂ S1+GSNO	-2.830	-7.130 to 1.470	No	ns	0.3606
♂ Control vs. ♂ S1+N6022	-3.163	-7.463 to 1.137	No	ns	0.2443
♂ Control vs. ♀ Control	-0.09000	-4.390 to 4.210	No	ns	>0.9999
♂ Control vs. ♀ S1	-7.530	-11.83 to -3.230	Yes	***	0.0003
♂ Control vs. ♀ S1+GSNO	-3.163	-7.463 to 1.137	No	ns	0.2443
♂ Control vs. ♀ S1+N6022	-4.030	-8.330 to 0.2701	No	ns	0.0747
♂ S1 vs. ♂ S1+GSNO	10.90	6.600 to 15.20	Yes	****	<0.0001
♂ S1 vs. ♂ S1+N6022	10.57	6.267 to 14.87	Yes	****	<0.0001
♂ S1 vs. ♀ Control	13.64	9.340 to 17.94	Yes	****	<0.0001
♂ S1 vs. ♀ S1	6.200	1.900 to 10.50	Yes	**	0.0026
♂ S1 vs. ♀ S1+GSNO	10.57	6.267 to 14.87	Yes	****	<0.0001
♂ S1 vs. ♀ S1+N6022	9.700	5.400 to 14.00	Yes	****	<0.0001
♂ S1+GSNO vs. ♂ S1+N6022	-0.3333	-4.633 to 3.967	No	ns	>0.9999
♂ S1+GSNO vs. ♀ Control	2.740	-1.560 to 7.040	No	ns	0.3971
♂ S1+GSNO vs. ♀ S1	-4.700	-9.000 to -0.3999	Yes	*	0.0271
♂ S1+GSNO vs. ♀ S1+GSNO	-0.3333	-4.633 to 3.967	No	ns	>0.9999
♂ S1+GSNO vs. ♀ S1+N6022	-1.200	-5.500 to 3.100	No	ns	0.9732
♂ S1+N6022 vs. ♀ Control	3.073	-1.227 to 7.373	No	ns	0.2726
♂ S1+N6022 vs. ♀ S1	-4.367	-8.667 to -0.06656	Yes	*	0.0452
♂ S1+N6022 vs. ♀ S1+GSNO	0.000	-4.300 to 4.300	No	ns	>0.9999
♂ S1+N6022 vs. ♀ S1+N6022	-0.8667	-5.167 to 3.433	No	ns	0.9959
♀ Control vs. ♀ S1	-7.440	-11.74 to -3.140	Yes	***	0.0004
♀ Control vs. ♀ S1+GSNO	-3.073	-7.373 to 1.227	No	ns	0.2726
♀ Control vs. ♀ S1+N6022	-3.940	-8.240 to 0.3601	No	ns	0.0852
♀ S1 vs. ♀ S1+GSNO	4.367	0.06656 to 8.667	Yes	*	0.0452
♀ S1 vs. ♀ S1+N6022	3.500	-0.8001 to 7.800	No	ns	0.1580
♀ S1+GSNO vs. ♀ S1+N6022	-0.8667	-5.167 to 3.433	No	ns	0.9959

Statistical Analysis of figure 3A-ii

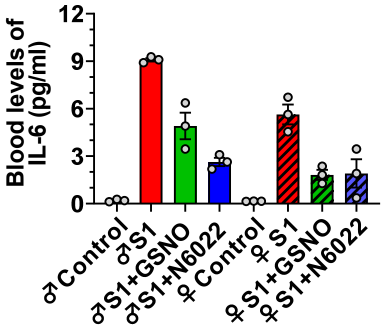

Tukey's multiple comparisons test	Mean Diff.	95.00% CI of diff.	Significant?	Summary	Adjusted P Value
♂ Control vs. ♂ S1	-8.896	-11.39 to -6.399	Yes	****	<0.0001
♂ Control vs. ♂ S1+GSNO	-4.712	-7.209 to -2.214	Yes	***	0.0001
♂ Control vs. ♂ S1+N6022	-2.441	-4.939 to 0.05597	No	ns	0.0578
♂ Control vs. ♀ Control	0.04100	-2.456 to 2.538	No	ns	>0.9999
♂ Control vs. ♀ S1	-5.441	-7.939 to -2.944	Yes	****	<0.0001
♂ Control vs. ♀ S1+GSNO	-1.623	-4.121 to 0.8742	No	ns	0.3746
♂ Control vs. ♀ S1+N6022	-1.714	-4.211 to 0.7832	No	ns	0.3146
♂ S1 vs. ♂ S1+GSNO	4.184	1.687 to 6.681	Yes	***	0.0006
♂ S1 vs. ♂ S1+N6022	6.455	3.957 to 8.952	Yes	****	<0.0001
♂ S1 vs. ♀ Control	8.937	6.440 to 11.43	Yes	****	<0.0001
♂ S1 vs. ♀ S1	3.455	0.9572 to 5.952	Yes	**	0.0038
♂ S1 vs. ♀ S1+GSNO	7.273	4.775 to 9.770	Yes	****	<0.0001
♂ S1 vs. ♀ S1+N6022	7.182	4.684 to 9.679	Yes	****	<0.0001
♂ S1+GSNO vs. ♂ S1+N6022	2.270	-0.2269 to 4.768	No	ns	0.0891
♂ S1+GSNO vs. ♀ Control	4.753	2.255 to 7.250	Yes	***	0.0001
♂ S1+GSNO vs. ♀ S1	-0.7295	-3.227 to 1.768	No	ns	0.9658
♂ S1+GSNO vs. ♀ S1+GSNO	3.089	0.5913 to 5.586	Yes	*	0.0103
♂ S1+GSNO vs. ♀ S1+N6022	2.998	0.5004 to 5.495	Yes	*	0.0132
♂ S1+N6022 vs. ♀ Control	2.482	-0.01497 to 4.980	No	ns	0.0520
♂ S1+N6022 vs. ♀ S1	-3.000	-5.497 to -0.5027	Yes	*	0.0131
♂ S1+N6022 vs. ♀ S1+GSNO	0.8182	-1.679 to 3.316	No	ns	0.9392
♂ S1+N6022 vs. ♀ S1+N6022	0.7273	-1.770 to 3.225	No	ns	0.9664
♀ Control vs. ♀ S1	-5.482	-7.980 to -2.985	Yes	****	<0.0001
♀ Control vs. ♀ S1+GSNO	-1.664	-4.162 to 0.8332	No	ns	0.3467
♀ Control vs. ♀ S1+N6022	-1.755	-4.252 to 0.7422	No	ns	0.2898
♀ S1 vs. ♀ S1+GSNO	3.818	1.321 to 6.316	Yes	**	0.0015
♀ S1 vs. ♀ S1+N6022	3.727	1.230 to 6.225	Yes	**	0.0019
♀ S1+GSNO vs. ♀ S1+N6022	-0.09091	-2.588 to 2.406	No	ns	>0.9999

Statistical Analysis of figure 3B-i

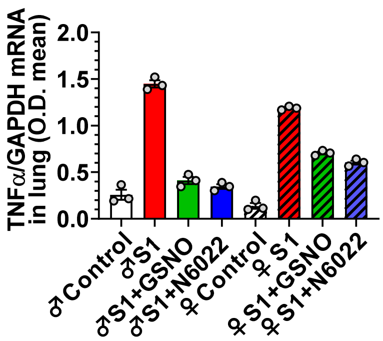

Tukey's multiple comparisons test	Mean Diff.	95.00% CI of diff.	Significant?	Summary	Adjusted P Value
♂ Control vs. ♂ S1	-1.189	-1.347 to -1.031	Yes	****	<0.0001
♂ Control vs. ♂ S1+GSNO	-0.1528	-0.3107 to 0.005101	No	ns	0.0616
♂ Control vs. ♂ S1+N6022	-0.08798	-0.2459 to 0.06991	No	ns	0.5521
♂ Control vs. ♀ Control	0.1199	-0.03799 to 0.2778	No	ns	0.2149
♂ Control vs. ♀ S1	-0.9294	-1.087 to -0.7716	Yes	****	<0.0001
♂ Control vs. ♀ S1+GSNO	-0.4479	-0.6058 to -0.2900	Yes	****	<0.0001
♂ Control vs. ♀ S1+N6022	-0.3455	-0.5034 to -0.1876	Yes	****	<0.0001
♂ S1 vs. ♂ S1+GSNO	1.037	0.8787 to 1.194	Yes	****	<0.0001
♂ S1 vs. ♂ S1+N6022	1.101	0.9435 to 1.259	Yes	****	<0.0001
♂ S1 vs. ♀ Control	1.309	1.151 to 1.467	Yes	****	<0.0001
♂ S1 vs. ♀ S1	0.2599	0.1020 to 0.4178	Yes	***	0.0007
♂ S1 vs. ♀ S1+GSNO	0.7414	0.5835 to 0.8993	Yes	****	<0.0001
♂ S1 vs. ♀ S1+N6022	0.8439	0.6860 to 1.002	Yes	****	<0.0001
♂ S1+GSNO vs. ♂ S1+N6022	0.06481	-0.09308 to 0.2227	No	ns	0.8351
♂ S1+GSNO vs. ♀ Control	0.2727	0.1148 to 0.4306	Yes	***	0.0004
♂ S1+GSNO vs. ♀ S1	-0.7767	-0.9345 to -0.6188	Yes	****	<0.0001
♂ S1+GSNO vs. ♀ S1+GSNO	-0.2951	-0.4530 to -0.1373	Yes	***	0.0002
♂ S1+GSNO vs. ♀ S1+N6022	-0.1927	-0.3506 to -0.03482	Yes	*	0.0115
♂ S1+N6022 vs. ♀ Control	0.2079	0.04999 to 0.3658	Yes	**	0.0060
♂ S1+N6022 vs. ♀ S1	-0.8415	-0.9993 to -0.6836	Yes	****	<0.0001
♂ S1+N6022 vs. ♀ S1+GSNO	-0.3600	-0.5178 to -0.2021	Yes	****	<0.0001
♂ S1+N6022 vs. ♀ S1+N6022	-0.2575	-0.4154 to -0.09963	Yes	***	0.0007
♀ Control vs. ♀ S1	-1.049	-1.207 to -0.8914	Yes	****	<0.0001
♀ Control vs. ♀ S1+GSNO	-0.5678	-0.7257 to -0.4099	Yes	****	<0.0001
♀ Control vs. ♀ S1+N6022	-0.4654	-0.6233 to -0.3075	Yes	****	<0.0001
♀ S1 vs. ♀ S1+GSNO	0.4815	0.3236 to 0.6394	Yes	****	<0.0001
♀ S1 vs. ♀ S1+N6022	0.5840	0.4261 to 0.7418	Yes	****	<0.0001
♀ S1+GSNO vs. ♀ S1+N6022	0.1024	-0.05544 to 0.2603	No	ns	0.3765

Statistical Analysis of figure 3B-ii

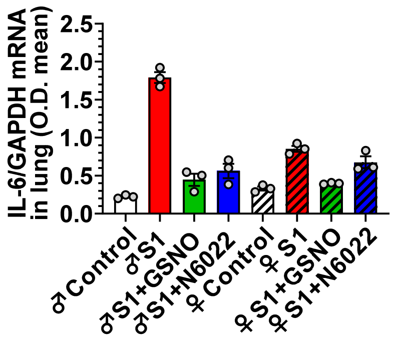

Tukey's multiple comparisons test	Mean Diff.	95.00% CI of diff.	Significant?	Summary	Adjusted P Value
♂ Control vs.♂ S1	-1.568	-1.863 to -1.273	Yes	****	<0.0001
♂ Control vs.♂ S1+GSNO	-0.2226	-0.5174 to 0.07211	No	ns	0.2197
♂ Control vs.♂ S1+N6022	-0.3396	-0.6343 to -0.04486	Yes	*	0.0183
♂ Control vs. ♀ Control	-0.1074	-0.4021 to 0.1873	No	ns	0.9002
♂ Control vs. ♀ S1	-0.6300	-0.9248 to -0.3353	Yes	****	<0.0001
♂ Control vs. ♀ S1+GSNO	-0.1736	-0.4684 to 0.1211	No	ns	0.4879
♂ Control vs. ♀ S1+N6022	-0.4533	-0.7480 to -0.1585	Yes	**	0.0014
♂ S1 vs.♂ S1+GSNO	1.345	1.051 to 1.640	Yes	****	<0.0001
♂ S1 vs.♂ S1+N6022	1.228	0.9337 to 1.523	Yes	****	<0.0001
♂ S1 vs. ♀ Control	1.461	1.166 to 1.755	Yes	****	<0.0001
♂ S1 vs. ♀ S1	0.9380	0.6433 to 1.233	Yes	****	<0.0001
♂ S1 vs. ♀ S1+GSNO	1.394	1.100 to 1.689	Yes	****	<0.0001
♂ S1 vs. ♀ S1+N6022	1.115	0.8200 to 1.410	Yes	****	<0.0001
♂ S1+GSNO vs.♂ S1+N6022	-0.1170	-0.4117 to 0.1778	No	ns	0.8561
♂ S1+GSNO vs. ♀ Control	0.1152	-0.1795 to 0.4100	No	ns	0.8648
♂ S1+GSNO vs. ♀ S1	-0.4074	-0.7021 to -0.1127	Yes	**	0.0039
♂ S1+GSNO vs. ♀ S1+GSNO	0.04898	-0.2458 to 0.3437	No	ns	0.9987
♂ S1+GSNO vs. ♀ S1+N6022	-0.2306	-0.5254 to 0.06411	No	ns	0.1890
♂ S1+N6022 vs. ♀ Control	0.2322	-0.06253 to 0.5269	No	ns	0.1834
♂ S1+N6022 vs. ♀ S1	-0.2904	-0.5852 to 0.004316	No	ns	0.0550
♂ S1+N6022 vs. ♀ S1+GSNO	0.1660	-0.1288 to 0.4607	No	ns	0.5402
♂ S1+N6022 vs. ♀ S1+N6022	-0.1137	-0.4084 to 0.1811	No	ns	0.8724
♀ Control vs. ♀ S1	-0.5226	-0.8174 to -0.2279	Yes	***	0.0003
♀ Control vs. ♀ S1+GSNO	-0.06625	-0.3610 to 0.2285	No	ns	0.9920
♀ Control vs. ♀ S1+N6022	-0.3459	-0.6406 to -0.05113	Yes	*	0.0158
♀ S1 vs. ♀ S1+GSNO	0.4564	0.1616 to 0.7511	Yes	**	0.0013
♀ S1 vs. ♀ S1+N6022	0.1768	-0.1180 to 0.4715	No	ns	0.4672
♀ S1+GSNO vs. ♀ S1+N6022	-0.2796	-0.5744 to 0.01512	No	ns	0.0695

Statistical Analysis of figure 3B-iii

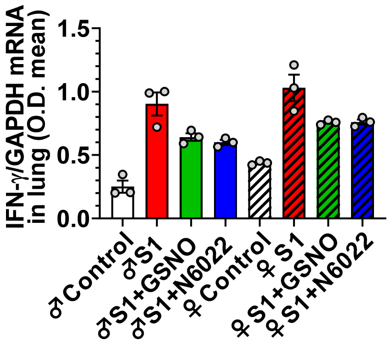

Tukey's multiple comparisons test	Mean Diff.	95.00% CI of diff.	Significant?	Summary	Adjusted P Value
♂ Control vs. ♂ S1	-0.6523	-0.9182 to -0.3864	Yes	****	<0.0001
♂ Control vs. ♂ S1+GSNO	-0.3910	-0.6569 to -0.1251	Yes	**	0.0021
♂ Control vs. ♂ S1+N6022	-0.3480	-0.6139 to -0.08213	Yes	**	0.0063
♂ Control vs. ♀ Control	-0.1864	-0.4522 to 0.07951	No	ns	0.2925
♂ Control vs. ♀ S1	-0.7778	-1.044 to -0.5120	Yes	****	<0.0001
♂ Control vs. ♀ S1+GSNO	-0.5066	-0.7725 to -0.2408	Yes	***	0.0001
♂ Control vs. ♀ S1+N6022	-0.5090	-0.7749 to -0.2431	Yes	***	0.0001
♂ S1 vs. ♂ S1+GSNO	0.2613	-0.004608 to 0.5271	No	ns	0.0559
♂ S1 vs. ♂ S1+N6022	0.3043	0.03841 to 0.5702	Yes	*	0.0192
♂ S1 vs. ♀ Control	0.4659	0.2000 to 0.7318	Yes	***	0.0003
♂ S1 vs. ♀ S1	-0.1255	-0.3914 to 0.1403	No	ns	0.7248
♂ S1 vs. ♀ S1+GSNO	0.1457	-0.1202 to 0.4115	No	ns	0.5713
♂ S1 vs. ♀ S1+N6022	0.1433	-0.1226 to 0.4091	No	ns	0.5898
♂ S1+GSNO vs. ♂ S1+N6022	0.04302	-0.2229 to 0.3089	No	ns	0.9989
♂ S1+GSNO vs. ♀ Control	0.2047	-0.06122 to 0.4705	No	ns	0.2030
♂ S1+GSNO vs. ♀ S1	-0.3868	-0.6527 to -0.1209	Yes	**	0.0024
♂ S1+GSNO vs. ♀ S1+GSNO	-0.1156	-0.3815 to 0.1503	No	ns	0.7942
♂ S1+GSNO vs. ♀ S1+N6022	-0.1180	-0.3839 to 0.1479	No	ns	0.7782
♂ S1+N6022 vs. ♀ Control	0.1616	-0.1042 to 0.4275	No	ns	0.4514
♂ S1+N6022 vs. ♀ S1	-0.4298	-0.6957 to -0.1640	Yes	***	0.0008
♂ S1+N6022 vs. ♀ S1+GSNO	-0.1586	-0.4245 to 0.1072	No	ns	0.4732
♂ S1+N6022 vs. ♀ S1+N6022	-0.1610	-0.4269 to 0.1049	No	ns	0.4559
♀ Control vs. ♀ S1	-0.5915	-0.8573 to -0.3256	Yes	****	<0.0001
♀ Control vs. ♀ S1+GSNO	-0.3203	-0.5861 to -0.05439	Yes	*	0.0128
♀ Control vs. ♀ S1+N6022	-0.3227	-0.5885 to -0.05678	Yes	*	0.0121
♀ S1 vs. ♀ S1+GSNO	0.2712	0.005325 to 0.5371	Yes	*	0.0439
♀ S1 vs. ♀ S1+N6022	0.2688	0.002937 to 0.5347	Yes	*	0.0465
♀ S1+GSNO vs. ♀ S1+N6022	-0.002387	-0.2683 to 0.2635	No	ns	>0.9999

Statistical Analysis of figure 3B-iv

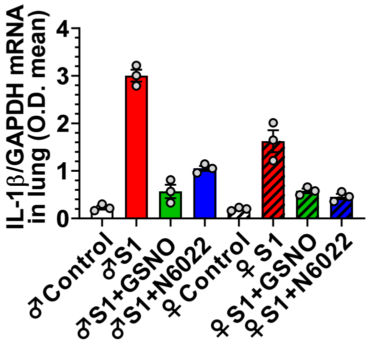

Tukey's multiple comparisons test	Mean Diff.	95.00% CI of diff.	Significant?	Summary	Adjusted P Value
♂ Control vs.♂ S1	-2.776	-3.320 to -2.232	Yes	****	<0.0001
♂ Control vs.♂ S1+GSNO	-0.3418	-0.8855 to 0.2019	No	ns	0.4126
♂ Control vs.♂ S1+N6022	-0.8238	-1.368 to -0.2801	Yes	**	0.0016
♂ Control vs. ♀ Control	0.02892	-0.5148 to 0.5726	No	ns	>0.9999
♂ Control vs. ♀ S1	-1.398	-1.942 to -0.8542	Yes	****	<0.0001
♂ Control vs. ♀ S1+GSNO	-0.3521	-0.8958 to 0.1916	No	ns	0.3788
♂ Control vs. ♀ S1+N6022	-0.2321	-0.7758 to 0.3116	No	ns	0.8081
♂ S1 vs.♂ S1+GSNO	2.434	1.891 to 2.978	Yes	****	<0.0001
♂ S1 vs.♂ S1+N6022	1.952	1.409 to 2.496	Yes	****	<0.0001
♂ S1 vs. ♀ Control	2.805	2.261 to 3.349	Yes	****	<0.0001
♂ S1 vs. ♀ S1	1.378	0.8344 to 1.922	Yes	****	<0.0001
♂ S1 vs. ♀ S1+GSNO	2.424	1.880 to 2.968	Yes	****	<0.0001
♂ S1 vs. ♀ S1+N6022	2.544	2.000 to 3.088	Yes	****	<0.0001
♂ S1+GSNO vs.♂ S1+N6022	-0.4820	-1.026 to 0.06174	No	ns	0.1025
♂ S1+GSNO vs. ♀ Control	0.3707	-0.1730 to 0.9144	No	ns	0.3217
♂ S1+GSNO vs. ♀ S1	-1.056	-1.600 to -0.5124	Yes	***	0.0001
♂ S1+GSNO vs. ♀ S1+GSNO	-0.01029	-0.5540 to 0.5334	No	ns	>0.9999
♂ S1+GSNO vs. ♀ S1+N6022	0.1097	-0.4340 to 0.6534	No	ns	0.9958
♂ S1+N6022 vs. ♀ Control	0.8527	0.3090 to 1.396	Yes	**	0.0011
♂ S1+N6022 vs. ♀ S1	-0.5742	-1.118 to -0.03046	Yes	*	0.0347
♂ S1+N6022 vs. ♀ S1+GSNO	0.4717	-0.07203 to 1.015	No	ns	0.1150
♂ S1+N6022 vs. ♀ S1+N6022	0.5917	0.04798 to 1.135	Yes	*	0.0280
♀ Control vs. ♀ S1	-1.427	-1.971 to -0.8832	Yes	****	<0.0001
♀ Control vs. ♀ S1+GSNO	-0.3810	-0.9247 to 0.1627	No	ns	0.2928
♀ Control vs. ♀ S1+N6022	-0.2610	-0.8047 to 0.2827	No	ns	0.7094
♀ S1 vs. ♀ S1+GSNO	1.046	0.5022 to 1.590	Yes	***	0.0001
♀ S1 vs. ♀ S1+N6022	1.166	0.6222 to 1.710	Yes	****	<0.0001
♀ S1+GSNO vs. ♀ S1+N6022	0.1200	-0.4237 to 0.6637	No	ns	0.9929

Statistical Analysis of figure 4A-i

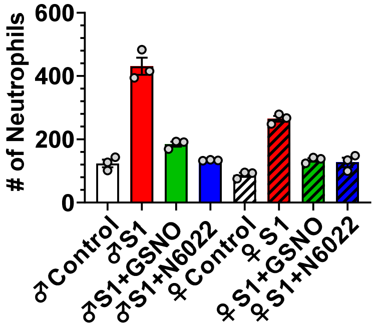

Tukey's multiple comparisons test	Mean Diff.	95.00% CI of diff.	Significant?	Summary	Adjusted P Value
♂ Control vs. ♂ S1	-307.0	-369.3 to -244.7	Yes	****	<0.0001
♂ Control vs. ♂ S1+GSNO	-60.67	-123.0 to 1.631	No	ns	0.0592
♂ Control vs. ♂ S1+N6022	-9.667	-71.96 to 52.63	No	ns	0.9992
♂ Control vs. ♀ Control	36.00	-26.30 to 98.30	No	ns	0.5103
♂ Control vs. ♀ S1	-141.0	-203.3 to -78.70	Yes	****	<0.0001
♂ Control vs. ♀ S1+GSNO	-11.00	-73.30 to 51.30	No	ns	0.9982
♂ Control vs. ♀ S1+N6022	-3.333	-65.63 to 58.96	No	ns	>0.9999
♂ S1 vs. ♂ S1+GSNO	246.3	184.0 to 308.6	Yes	****	<0.0001
♂ S1 vs. ♂ S1+N6022	297.3	235.0 to 359.6	Yes	****	<0.0001
♂ S1 vs. ♀ Control	343.0	280.7 to 405.3	Yes	****	<0.0001
♂ S1 vs. ♀ S1	166.0	103.7 to 228.3	Yes	****	<0.0001
♂ S1 vs. ♀ S1+GSNO	296.0	233.7 to 358.3	Yes	****	<0.0001
♂ S1 vs. ♀ S1+N6022	303.7	241.4 to 366.0	Yes	****	<0.0001
♂ S1+GSNO vs. ♂ S1+N6022	51.00	-11.30 to 113.3	No	ns	0.1537
♂ S1+GSNO vs. ♀ Control	96.67	34.37 to 159.0	Yes	**	0.0013
♂ S1+GSNO vs. ♀ S1	-80.33	-142.6 to -18.04	Yes	**	0.0072
♂ S1+GSNO vs. ♀ S1+GSNO	49.67	-12.63 to 112.0	No	ns	0.1739
♂ S1+GSNO vs. ♀ S1+N6022	57.33	-4.964 to 119.6	No	ns	0.0831
♂ S1+N6022 vs. ♀ Control	45.67	-16.63 to 108.0	No	ns	0.2477
♂ S1+N6022 vs. ♀ S1	-131.3	-193.6 to -69.04	Yes	****	<0.0001
♂ S1+N6022 vs. ♀ S1+GSNO	-1.333	-63.63 to 60.96	No	ns	>0.9999
♂ S1+N6022 vs. ♀ S1+N6022	6.333	-55.96 to 68.63	No	ns	>0.9999
♀ Control vs. ♀ S1	-177.0	-239.3 to -114.7	Yes	****	<0.0001
♀ Control vs. ♀ S1+GSNO	-47.00	-109.3 to 15.30	No	ns	0.2208
♀ Control vs. ♀ S1+N6022	-39.33	-101.6 to 22.96	No	ns	0.4076
♀ S1 vs. ♀ S1+GSNO	130.0	67.70 to 192.3	Yes	****	<0.0001
♀ S1 vs. ♀ S1+N6022	137.7	75.37 to 200.0	Yes	****	<0.0001
♀ S1+GSNO vs. ♀ S1+N6022	7.667	-54.63 to 69.96	No	ns	0.9998

Statistical Analysis of figure 4A-ii

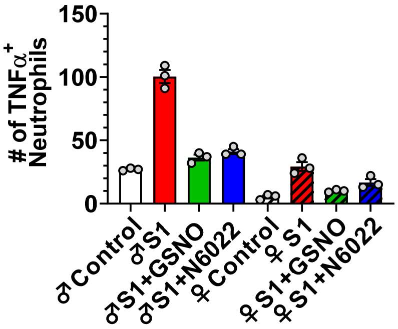

Tukey's multiple comparisons test	Mean Diff.	95.00% CI of diff.	Significant?	Summary	Adjusted P Value
♂ Control vs. ♂ S1	-73.33	-86.58 to -60.09	Yes	****	<0.0001
♂ Control vs. ♂ S1+GSNO	-9.333	-22.58 to 3.913	No	ns	0.2871
♂ Control vs. ♂ S1+N6022	-14.00	-27.25 to -0.7535	Yes	*	0.0345
♂ Control vs. ♀ Control	21.67	8.420 to 34.91	Yes	***	0.0007
♂ Control vs. ♀ S1	-2.333	-15.58 to 10.91	No	ns	0.9982
♂ Control vs. ♀ S1+GSNO	17.00	3.754 to 30.25	Yes	**	0.0075
♂ Control vs. ♀ S1+N6022	10.33	-2.913 to 23.58	No	ns	0.1916
♂ S1 vs. ♂ S1+GSNO	64.00	50.75 to 77.25	Yes	****	<0.0001
♂ S1 vs. ♂ S1+N6022	59.33	46.09 to 72.58	Yes	****	<0.0001
♂ S1 vs. ♀ Control	95.00	81.75 to 108.2	Yes	****	<0.0001
♂ S1 vs. ♀ S1	71.00	57.75 to 84.25	Yes	****	<0.0001
♂ S1 vs. ♀ S1+GSNO	90.33	77.09 to 103.6	Yes	****	<0.0001
♂ S1 vs. ♀ S1+N6022	83.67	70.42 to 96.91	Yes	****	<0.0001
♂ S1+GSNO vs. ♂ S1+N6022	-4.667	-17.91 to 8.580	No	ns	0.9143
♂ S1+GSNO vs. ♀ Control	31.00	17.75 to 44.25	Yes	****	<0.0001
♂ S1+GSNO vs. ♀ S1	7.000	-6.246 to 20.25	No	ns	0.6112
♂ S1+GSNO vs. ♀ S1+GSNO	26.33	13.09 to 39.58	Yes	****	<0.0001
♂ S1+GSNO vs. ♀ S1+N6022	19.67	6.420 to 32.91	Yes	**	0.0020
♂ S1+N6022 vs. ♀ Control	35.67	22.42 to 48.91	Yes	****	<0.0001
♂ S1+N6022 vs. ♀ S1	11.67	-1.580 to 24.91	No	ns	0.1061
♂ S1+N6022 vs. ♀ S1+GSNO	31.00	17.75 to 44.25	Yes	****	<0.0001
♂ S1+N6022 vs. ♀ S1+N6022	24.33	11.09 to 37.58	Yes	***	0.0002
♀ Control vs. ♀ S1	-24.00	-37.25 to -10.75	Yes	***	0.0002
♀ Control vs. ♀ S1+GSNO	-4.667	-17.91 to 8.580	No	ns	0.9143
♀ Control vs. ♀ S1+N6022	-11.33	-24.58 to 1.913	No	ns	0.1236
♀ S1 vs. ♀ S1+GSNO	19.33	6.087 to 32.58	Yes	**	0.0023
♀ S1 vs. ♀ S1+N6022	12.67	-0.5798 to 25.91	No	ns	0.0662
♀ S1+GSNO vs. ♀ S1+N6022	-6.667	-19.91 to 6.580	No	ns	0.6628

Statistical Analysis of figure 4B-i

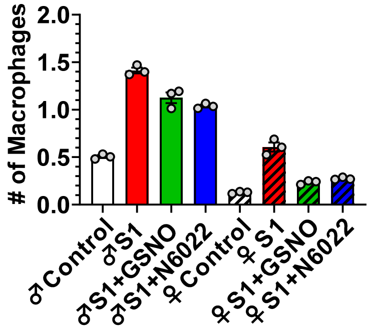

Tukey's multiple comparisons test	Mean Diff.	95.00% CI of diff.	Significant?	Summary	Adjusted P Value
♂ Control vs.♂ S1	-0.9070	-1.052 to -0.7615	Yes	****	<0.0001
♂ Control vs.♂ S1+GSNO	-0.6203	-0.7658 to -0.4749	Yes	****	<0.0001
♂ Control vs.♂ S1+N6022	-0.5330	-0.6785 to -0.3875	Yes	****	<0.0001
♂ Control vs. ♀ Control	0.3783	0.2329 to 0.5238	Yes	****	<0.0001
♂ Control vs. ♀ S1	-0.1003	-0.2458 to 0.04513	No	ns	0.3094
♂ Control vs. ♀ S1+GSNO	0.2707	0.1252 to 0.4161	Yes	***	0.0002
♂ Control vs. ♀ S1+N6022	0.2310	0.08554 to 0.3765	Yes	***	0.0010
♂ S1 vs.♂ S1+GSNO	0.2867	0.1412 to 0.4321	Yes	****	<0.0001
♂ S1 vs.♂ S1+N6022	0.3740	0.2285 to 0.5195	Yes	****	<0.0001
♂ S1 vs. ♀ Control	1.285	1.140 to 1.431	Yes	****	<0.0001
♂ S1 vs. ♀ S1	0.8067	0.6612 to 0.9521	Yes	****	<0.0001
♂ S1 vs. ♀ S1+GSNO	1.178	1.032 to 1.323	Yes	****	<0.0001
♂ S1 vs. ♀ S1+N6022	1.138	0.9925 to 1.283	Yes	****	<0.0001
♂ S1+GSNO vs.♂ S1+N6022	0.08733	-0.05813 to 0.2328	No	ns	0.4659
♂ S1+GSNO vs. ♀ Control	0.9987	0.8532 to 1.144	Yes	****	<0.0001
♂ S1+GSNO vs. ♀ S1	0.5200	0.3745 to 0.6655	Yes	****	<0.0001
♂ S1+GSNO vs. ♀ S1+GSNO	0.8910	0.7455 to 1.036	Yes	****	<0.0001
♂ S1+GSNO vs. ♀ S1+N6022	0.8513	0.7059 to 0.9968	Yes	****	<0.0001
♂ S1+N6022 vs. ♀ Control	0.9113	0.7659 to 1.057	Yes	****	<0.0001
♂ S1+N6022 vs. ♀ S1	0.4327	0.2872 to 0.5781	Yes	****	<0.0001
♂ S1+N6022 vs. ♀ S1+GSNO	0.8037	0.6582 to 0.9491	Yes	****	<0.0001
♂ S1+N6022 vs. ♀ S1+N6022	0.7640	0.6185 to 0.9095	Yes	****	<0.0001
♀ Control vs. ♀ S1	-0.4787	-0.6241 to -0.3332	Yes	****	<0.0001
♀ Control vs. ♀ S1+GSNO	-0.1077	-0.2531 to 0.03779	No	ns	0.2384
♀ Control vs. ♀ S1+N6022	-0.1473	-0.2928 to -0.001873	Yes	*	0.0460
♀ S1 vs. ♀ S1+GSNO	0.3710	0.2255 to 0.5165	Yes	****	<0.0001
♀ S1 vs. ♀ S1+N6022	0.3313	0.1859 to 0.4768	Yes	****	<0.0001
♀ S1+GSNO vs. ♀ S1+N6022	-0.03967	-0.1851 to 0.1058	No	ns	0.9763

Statistical Analysis of figure 4B-ii

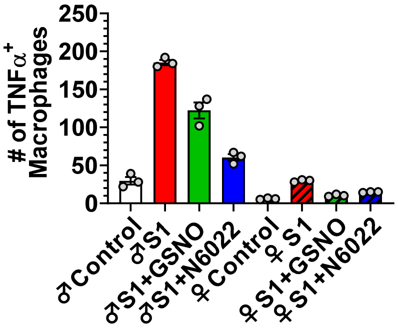

Tukey's multiple comparisons test	Mean Diff.	95.00% CI of diff.	Significant?	Summary	Adjusted P Value
♂ Control vs. ♂ S1	-155.7	-178.2 to -133.2	Yes	****	<0.0001
♂ Control vs. ♂ S1+GSNO	-92.67	-115.2 to -70.18	Yes	****	<0.0001
♂ Control vs. ♂ S1+N6022	-30.67	-53.16 to -8.177	Yes	**	0.0044
♂ Control vs. ♀ Control	23.67	1.177 to 46.16	Yes	*	0.0355
♂ Control vs. ♀ S1	0.000	-22.49 to 22.49	No	ns	>0.9999
♂ Control vs. ♀ S1+GSNO	19.33	-3.156 to 41.82	No	ns	0.1206
♂ Control vs. ♀ S1+N6022	15.00	-7.489 to 37.49	No	ns	0.3457
♂ S1 vs. ♂ S1+GSNO	63.00	40.51 to 85.49	Yes	****	<0.0001
♂ S1 vs. ♂ S1+N6022	125.0	102.5 to 147.5	Yes	****	<0.0001
♂ S1 vs. ♀ Control	179.3	156.8 to 201.8	Yes	****	<0.0001
♂ S1 vs. ♀ S1	155.7	133.2 to 178.2	Yes	****	<0.0001
♂ S1 vs. ♀ S1+GSNO	175.0	152.5 to 197.5	Yes	****	<0.0001
♂ S1 vs. ♀ S1+N6022	170.7	148.2 to 193.2	Yes	****	<0.0001
♂ S1+GSNO vs. ♂ S1+N6022	62.00	39.51 to 84.49	Yes	****	<0.0001
♂ S1+GSNO vs. ♀ Control	116.3	93.84 to 138.8	Yes	****	<0.0001
♂ S1+GSNO vs. ♀ S1	92.67	70.18 to 115.2	Yes	****	<0.0001
♂ S1+GSNO vs. ♀ S1+GSNO	112.0	89.51 to 134.5	Yes	****	<0.0001
♂ S1+GSNO vs. ♀ S1+N6022	107.7	85.18 to 130.2	Yes	****	<0.0001
♂ S1+N6022 vs. ♀ Control	54.33	31.84 to 76.82	Yes	****	<0.0001
♂ S1+N6022 vs. ♀ S1	30.67	8.177 to 53.16	Yes	**	0.0044
♂ S1+N6022 vs. ♀ S1+GSNO	50.00	27.51 to 72.49	Yes	****	<0.0001
♂ S1+N6022 vs. ♀ S1+N6022	45.67	23.18 to 68.16	Yes	****	<0.0001
♀ Control vs. ♀ S1	-23.67	-46.16 to -1.177	Yes	*	0.0355
♀ Control vs. ♀ S1+GSNO	-4.333	-26.82 to 18.16	No	ns	0.9968
♀ Control vs. ♀ S1+N6022	-8.667	-31.16 to 13.82	No	ns	0.8727
♀ S1 vs. ♀ S1+GSNO	19.33	-3.156 to 41.82	No	ns	0.1206
♀ S1 vs. ♀ S1+N6022	15.00	-7.489 to 37.49	No	ns	0.3457
♀ S1+GSNO vs. ♀ S1+N6022	-4.333	-26.82 to 18.16	No	ns	0.9968

Statistical Analysis of figure 5A-i

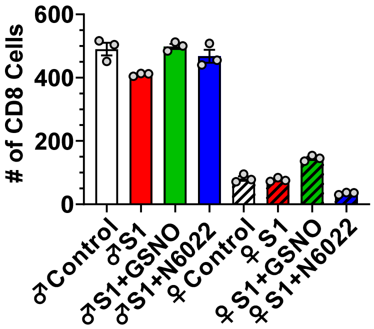

Tukey's multiple comparisons test	Mean Diff.	95.00% CI of diff.	Significant?	Summary	Adjusted P Value
♂ Control vs. ♂ S1	80.67	26.10 to 135.2	Yes	**	0.0020
♂ Control vs. ♂ S1+GSNO	-8.333	-62.90 to 46.23	No	ns	0.9993
♂ Control vs. ♂ S1+N6022	22.67	-31.90 to 77.23	No	ns	0.8272
♂ Control vs. ♀ Control	409.0	354.4 to 463.6	Yes	****	<0.0001
♂ Control vs. ♀ S1	413.3	358.8 to 467.9	Yes	****	<0.0001
♂ Control vs. ♀ S1+GSNO	345.3	290.8 to 399.9	Yes	****	<0.0001
♂ Control vs. ♀ S1+N6022	456.0	401.4 to 510.6	Yes	****	<0.0001
♂ S1 vs. ♂ S1+GSNO	-89.00	-143.6 to -34.43	Yes	***	0.0007
♂ S1 vs. ♂ S1+N6022	-58.00	-112.6 to -3.432	Yes	*	0.0331
♂ S1 vs. ♀ Control	328.3	273.8 to 382.9	Yes	****	<0.0001
♂ S1 vs. ♀ S1	332.7	278.1 to 387.2	Yes	****	<0.0001
♂ S1 vs. ♀ S1+GSNO	264.7	210.1 to 319.2	Yes	****	<0.0001
♂ S1 vs. ♀ S1+N6022	375.3	320.8 to 429.9	Yes	****	<0.0001
♂ S1+GSNO vs. ♂ S1+N6022	31.00	-23.57 to 85.57	No	ns	0.5300
♂ S1+GSNO vs. ♀ Control	417.3	362.8 to 471.9	Yes	****	<0.0001
♂ S1+GSNO vs. ♀ S1	421.7	367.1 to 476.2	Yes	****	<0.0001
♂ S1+GSNO vs. ♀ S1+GSNO	353.7	299.1 to 408.2	Yes	****	<0.0001
♂ S1+GSNO vs. ♀ S1+N6022	464.3	409.8 to 518.9	Yes	****	<0.0001
♂ S1+N6022 vs. ♀ Control	386.3	331.8 to 440.9	Yes	****	<0.0001
♂ S1+N6022 vs. ♀ S1	390.7	336.1 to 445.2	Yes	****	<0.0001
♂ S1+N6022 vs. ♀ S1+GSNO	322.7	268.1 to 377.2	Yes	****	<0.0001
♂ S1+N6022 vs. ♀ S1+N6022	433.3	378.8 to 487.9	Yes	****	<0.0001
♀ Control vs. ♀ S1	4.333	-50.23 to 58.90	No	ns	>0.9999
♀ Control vs. ♀ S1+GSNO	-63.67	-118.2 to -9.099	Yes	*	0.0166
♀ Control vs. ♀ S1+N6022	47.00	-7.568 to 101.6	No	ns	0.1194
♀ S1 vs. ♀ S1+GSNO	-68.00	-122.6 to -13.43	Yes	**	0.0097
♀ S1 vs. ♀ S1+N6022	42.67	-11.90 to 97.23	No	ns	0.1897
♀ S1+GSNO vs. ♀ S1+N6022	110.7	56.10 to 165.2	Yes	****	<0.0001

Statistical Analysis of figure 5A-ii

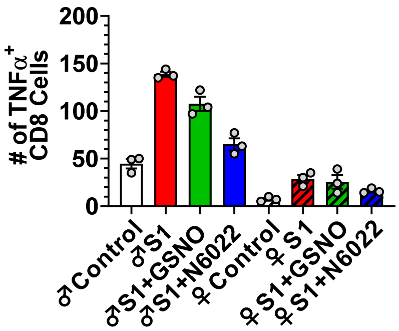

Tukey's multiple comparisons test	Mean Diff.	95.00% CI of diff.	Significant?	Summary	Adjusted P Value
♂ Control vs. ♂ S1	-94.00	-118.6 to -69.42	Yes	****	<0.0001
♂ Control vs. ♂ S1+GSNO	-63.00	-87.58 to -38.42	Yes	****	<0.0001
♂ Control vs. ♂ S1+N6022	-20.33	-44.92 to 4.250	No	ns	0.1463
♂ Control vs. ♀ Control	37.33	12.75 to 61.92	Yes	**	0.0016
♂ Control vs. ♀ S1	16.00	-8.583 to 40.58	No	ns	0.3730
♂ Control vs. ♀ S1+GSNO	19.00	-5.583 to 43.58	No	ns	0.1995
♂ Control vs. ♀ S1+N6022	28.67	4.084 to 53.25	Yes	*	0.0166
♂ S1 vs. ♂ S1+GSNO	31.00	6.417 to 55.58	Yes	**	0.0088
♂ S1 vs. ♂ S1+N6022	73.67	49.08 to 98.25	Yes	****	<0.0001
♂ S1 vs. ♀ Control	131.3	106.8 to 155.9	Yes	****	<0.0001
♂ S1 vs. ♀ S1	110.0	85.42 to 134.6	Yes	****	<0.0001
♂ S1 vs. ♀ S1+GSNO	113.0	88.42 to 137.6	Yes	****	<0.0001
♂ S1 vs. ♀ S1+N6022	122.7	98.08 to 147.2	Yes	****	<0.0001
♂ S1+GSNO vs. ♂ S1+N6022	42.67	18.08 to 67.25	Yes	***	0.0004
♂ S1+GSNO vs. ♀ Control	100.3	75.75 to 124.9	Yes	****	<0.0001
♂ S1+GSNO vs. ♀ S1	79.00	54.42 to 103.6	Yes	****	<0.0001
♂ S1+GSNO vs. ♀ S1+GSNO	82.00	57.42 to 106.6	Yes	****	<0.0001
♂ S1+GSNO vs. ♀ S1+N6022	91.67	67.08 to 116.2	Yes	****	<0.0001
♂ S1+N6022 vs. ♀ Control	57.67	33.08 to 82.25	Yes	****	<0.0001
♂ S1+N6022 vs. ♀ S1	36.33	11.75 to 60.92	Yes	**	0.0020
♂ S1+N6022 vs. ♀ S1+GSNO	39.33	14.75 to 63.92	Yes	***	0.0009
♂ S1+N6022 vs. ♀ S1+N6022	49.00	24.42 to 73.58	Yes	****	<0.0001
♀ Control vs. ♀ S1	-21.33	-45.92 to 3.250	No	ns	0.1148
♀ Control vs. ♀ S1+GSNO	-18.33	-42.92 to 6.250	No	ns	0.2314
♀ Control vs. ♀ S1+N6022	-8.667	-33.25 to 15.92	No	ns	0.9140
♀ S1 vs. ♀ S1+GSNO	3.000	-21.58 to 27.58	No	ns	0.9998
♀ S1 vs. ♀ S1+N6022	12.67	-11.92 to 37.25	No	ns	0.6383
♀ S1+GSNO vs. ♀ S1+N6022	9.667	-14.92 to 34.25	No	ns	0.8615

Statistical Analysis of figure 5B-i

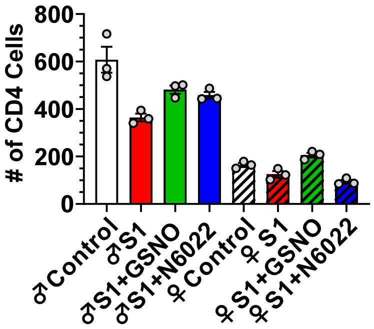

Tukey's multiple comparisons test	Mean Diff.	95.00% CI of diff.	Significant?	Summary	Adjusted P Value
♂ Control vs. ♂ S1	243.3	131.2 to 355.5	Yes	****	<0.0001
♂ Control vs. ♂ S1+GSNO	126.0	13.83 to 238.2	Yes	*	0.0222
♂ Control vs. ♂ S1+N6022	150.0	37.83 to 262.2	Yes	**	0.0052
♂ Control vs. ♀ Control	443.3	331.2 to 555.5	Yes	****	<0.0001
♂ Control vs. ♀ S1	483.0	370.8 to 595.2	Yes	****	<0.0001
♂ Control vs. ♀ S1+GSNO	402.0	289.8 to 514.2	Yes	****	<0.0001
♂ Control vs. ♀ S1+N6022	514.0	401.8 to 626.2	Yes	****	<0.0001
♂ S1 vs. ♂ S1+GSNO	-117.3	-229.5 to -5.166	Yes	*	0.0370
♂ S1 vs. ♂ S1+N6022	-93.33	-205.5 to 18.83	No	ns	0.1421
♂ S1 vs. ♀ Control	200.0	87.83 to 312.2	Yes	***	0.0003
♂ S1 vs. ♀ S1	239.7	127.5 to 351.8	Yes	****	<0.0001
♂ S1 vs. ♀ S1+GSNO	158.7	46.50 to 270.8	Yes	**	0.0031
♂ S1 vs. ♀ S1+N6022	270.7	158.5 to 382.8	Yes	****	<0.0001
♂ S1+GSNO vs. ♂ S1+N6022	24.00	-88.17 to 136.2	No	ns	0.9941
♂ S1+GSNO vs. ♀ Control	317.3	205.2 to 429.5	Yes	****	<0.0001
♂ S1+GSNO vs. ♀ S1	357.0	244.8 to 469.2	Yes	****	<0.0001
♂ S1+GSNO vs. ♀ S1+GSNO	276.0	163.8 to 388.2	Yes	****	<0.0001
♂ S1+GSNO vs. ♀ S1+N6022	388.0	275.8 to 500.2	Yes	****	<0.0001
♂ S1+N6022 vs. ♀ Control	293.3	181.2 to 405.5	Yes	****	<0.0001
♂ S1+N6022 vs. ♀ S1	333.0	220.8 to 445.2	Yes	****	<0.0001
♂ S1+N6022 vs. ♀ S1+GSNO	252.0	139.8 to 364.2	Yes	****	<0.0001
♂ S1+N6022 vs. ♀ S1+N6022	364.0	251.8 to 476.2	Yes	****	<0.0001
♀ Control vs. ♀ S1	39.67	-72.50 to 151.8	No	ns	0.9128
♀ Control vs. ♀ S1+GSNO	-41.33	-153.5 to 70.83	No	ns	0.8951
♀ Control vs. ♀ S1+N6022	70.67	-41.50 to 182.8	No	ns	0.4101
♀ S1 vs. ♀ S1+GSNO	-81.00	-193.2 to 31.17	No	ns	0.2623
♀ S1 vs. ♀ S1+N6022	31.00	-81.17 to 143.2	No	ns	0.9745
♀ S1+GSNO vs. ♀ S1+N6022	112.0	-0.1672 to 224.2	No	ns	0.0505

Statistical Analysis of figure 5B-ii

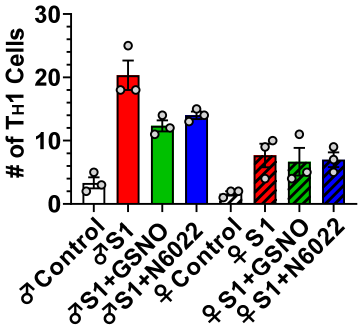

Tukey's multiple comparisons test	Mean Diff.	95.00% CI of diff.	Significant?	Summary	Adjusted P Value
♂ Control vs. ♂ S1	-17.00	-24.14 to -9.863	Yes	****	<0.0001
♂ Control vs. ♂ S1+GSNO	-9.000	-16.14 to -1.863	Yes	**	0.0088
♂ Control vs. ♂ S1+N6022	-10.67	-17.80 to -3.529	Yes	**	0.0018
♂ Control vs. ♀ Control	1.667	-5.471 to 8.804	No	ns	0.9901
♂ Control vs. ♀ S1	-4.333	-11.47 to 2.804	No	ns	0.4529
♂ Control vs. ♀ S1+GSNO	-3.333	-10.47 to 3.804	No	ns	0.7348
♂ Control vs. ♀ S1+N6022	-3.667	-10.80 to 3.471	No	ns	0.6414
♂ S1 vs. ♂ S1+GSNO	8.000	0.8626 to 15.14	Yes	*	0.0225
♂ S1 vs. ♂ S1+N6022	6.333	-0.8041 to 13.47	No	ns	0.1019
♂ S1 vs. ♀ Control	18.67	11.53 to 25.80	Yes	****	<0.0001
♂ S1 vs. ♀ S1	12.67	5.529 to 19.80	Yes	***	0.0003
♂ S1 vs. ♀ S1+GSNO	13.67	6.529 to 20.80	Yes	***	0.0001
♂ S1 vs. ♀ S1+N6022	13.33	6.196 to 20.47	Yes	***	0.0002
♂ S1+GSNO vs. ♂ S1+N6022	-1.667	-8.804 to 5.471	No	ns	0.9901
♂ S1+GSNO vs. ♀ Control	10.67	3.529 to 17.80	Yes	**	0.0018
♂ S1+GSNO vs. ♀ S1	4.667	-2.471 to 11.80	No	ns	0.3679
♂ S1+GSNO vs. ♀ S1+GSNO	5.667	-1.471 to 12.80	No	ns	0.1772
♂ S1+GSNO vs. ♀ S1+N6022	5.333	-1.804 to 12.47	No	ns	0.2296
♂ S1+N6022 vs. ♀ Control	12.33	5.196 to 19.47	Yes	***	0.0004
♂ S1+N6022 vs. ♀ S1	6.333	-0.8041 to 13.47	No	ns	0.1019
♂ S1+N6022 vs. ♀ S1+GSNO	7.333	0.1959 to 14.47	Yes	*	0.0418
♂ S1+N6022 vs. ♀ S1+N6022	7.000	-0.1374 to 14.14	No	ns	0.0566
♀ Control vs. ♀ S1	-6.000	-13.14 to 1.137	No	ns	0.1351
♀ Control vs. ♀ S1+GSNO	-5.000	-12.14 to 2.137	No	ns	0.2931
♀ Control vs. ♀ S1+N6022	-5.333	-12.47 to 1.804	No	ns	0.2296
♀ S1 vs. ♀ S1+GSNO	1.000	-6.137 to 8.137	No	ns	0.9996
♀ S1 vs. ♀ S1+N6022	0.6667	-6.471 to 7.804	No	ns	>0.9999
♀ S1+GSNO vs. ♀ S1+N6022	-0.3333	-7.471 to 6.804	No	ns	>0.9999

Statistical Analysis of figure 5B-iii

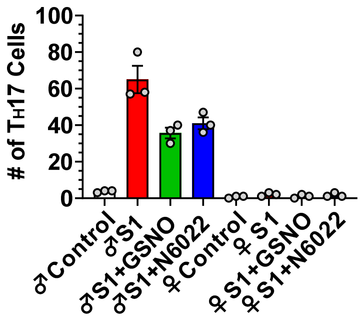

Tukey's multiple comparisons test	Mean Diff.	95.00% CI of diff.	Significant?	Summary	Adjusted P Value
♂ Control vs. ♂ S1	-61.33	-76.50 to -46.17	Yes	****	<0.0001
♂ Control vs. ♂ S1+GSNO	-32.00	-47.17 to -16.83	Yes	****	<0.0001
♂ Control vs. ♂ S1+N6022	-37.33	-52.50 to -22.17	Yes	****	<0.0001
♂ Control vs. ♀ Control	3.000	-12.17 to 18.17	No	ns	0.9963
♂ Control vs. ♀ S1	1.667	-13.50 to 16.83	No	ns	>0.9999
♂ Control vs. ♀ S1+GSNO	2.667	-12.50 to 17.83	No	ns	0.9982
♂ Control vs. ♀ S1+N6022	2.000	-13.17 to 17.17	No	ns	0.9997
♂ S1 vs. ♂ S1+GSNO	29.33	14.17 to 44.50	Yes	***	0.0001
♂ S1 vs. ♂ S1+N6022	24.00	8.832 to 39.17	Yes	**	0.0010
♂ S1 vs. ♀ Control	64.33	49.17 to 79.50	Yes	****	<0.0001
♂ S1 vs. ♀ S1	63.00	47.83 to 78.17	Yes	****	<0.0001
♂ S1 vs. ♀ S1+GSNO	64.00	48.83 to 79.17	Yes	****	<0.0001
♂ S1 vs. ♀ S1+N6022	63.33	48.17 to 78.50	Yes	****	<0.0001
♂ S1+GSNO vs. ♂ S1+N6022	-5.333	-20.50 to 9.835	No	ns	0.9151
♂ S1+GSNO vs. ♀ Control	35.00	19.83 to 50.17	Yes	****	<0.0001
♂ S1+GSNO vs. ♀ S1	33.67	18.50 to 48.83	Yes	****	<0.0001
♂ S1+GSNO vs. ♀ S1+GSNO	34.67	19.50 to 49.83	Yes	****	<0.0001
♂ S1+GSNO vs. ♀ S1+N6022	34.00	18.83 to 49.17	Yes	****	<0.0001
♂ S1+N6022 vs. ♀ Control	40.33	25.17 to 55.50	Yes	****	<0.0001
♂ S1+N6022 vs. ♀ S1	39.00	23.83 to 54.17	Yes	****	<0.0001
♂ S1+N6022 vs. ♀ S1+GSNO	40.00	24.83 to 55.17	Yes	****	<0.0001
♂ S1+N6022 vs. ♀ S1+N6022	39.33	24.17 to 54.50	Yes	****	<0.0001
♀ Control vs. ♀ S1	-1.333	-16.50 to 13.83	No	ns	>0.9999
♀ Control vs. ♀ S1+GSNO	-0.3333	-15.50 to 14.83	No	ns	>0.9999
♀ Control vs. ♀ S1+N6022	-1.000	-16.17 to 14.17	No	ns	>0.9999
♀ S1 vs. ♀ S1+GSNO	1.000	-14.17 to 16.17	No	ns	>0.9999
♀ S1 vs. ♀ S1+N6022	0.3333	-14.83 to 15.50	No	ns	>0.9999
♀ S1+GSNO vs. ♀ S1+N6022	-0.6667	-15.83 to 14.50	No	ns	>0.9999

Statistical Analysis of figure 6A-ii

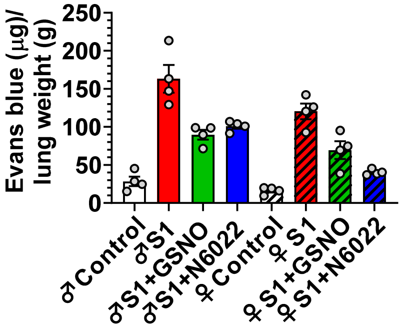

Tukey's multiple comparisons test	Mean Diff.	95.00% CI of diff.	Significant?	Summary	Adjusted P Value
♂ Control vs. ♂ S1	-134.8	-177.7 to -91.93	Yes	****	<0.0001
♂ Control vs. ♂ S1+GSNO	-61.24	-104.1 to -18.37	Yes	**	0.0018
♂ Control vs. ♂ S1+N6022	-72.37	-115.2 to -29.49	Yes	***	0.0002
♂ Control vs. ♀ Control	12.29	-30.58 to 55.16	No	ns	0.9773
♂ Control vs. ♀ S1	-91.92	-134.8 to -49.05	Yes	****	<0.0001
♂ Control vs. ♀ S1+GSNO	-41.06	-83.93 to 1.816	No	ns	0.0674
♂ Control vs. ♀ S1+N6022	-11.83	-54.70 to 31.04	No	ns	0.9817
♂ S1 vs. ♂ S1+GSNO	73.56	30.69 to 116.4	Yes	***	0.0002
♂ S1 vs. ♂ S1+N6022	62.44	19.57 to 105.3	Yes	**	0.0014
♂ S1 vs. ♀ Control	147.1	104.2 to 190.0	Yes	****	<0.0001
♂ S1 vs. ♀ S1	42.88	0.008573 to 85.75	Yes	*	0.0499
♂ S1 vs. ♀ S1+GSNO	93.75	50.88 to 136.6	Yes	****	<0.0001
♂ S1 vs. ♀ S1+N6022	123.0	80.10 to 165.8	Yes	****	<0.0001
♂ S1+GSNO vs. ♂ S1+N6022	-11.13	-54.00 to 31.74	No	ns	0.9871
♂ S1+GSNO vs. ♀ Control	73.53	30.66 to 116.4	Yes	***	0.0002
♂ S1+GSNO vs. ♀ S1	-30.69	-73.56 to 12.19	No	ns	0.2992
♂ S1+GSNO vs. ♀ S1+GSNO	20.18	-22.69 to 63.05	No	ns	0.7686
♂ S1+GSNO vs. ♀ S1+N6022	49.40	6.533 to 92.28	Yes	*	0.0161
♂ S1+N6022 vs. ♀ Control	84.66	41.79 to 127.5	Yes	****	<0.0001
♂ S1+N6022 vs. ♀ S1	-19.56	-62.43 to 23.31	No	ns	0.7944
♂ S1+N6022 vs. ♀ S1+GSNO	31.31	-11.56 to 74.18	No	ns	0.2771
♂ S1+N6022 vs. ♀ S1+N6022	60.53	17.66 to 103.4	Yes	**	0.0021
♀ Control vs. ♀ S1	-104.2	-147.1 to -61.34	Yes	****	<0.0001
♀ Control vs. ♀ S1+GSNO	-53.35	-96.22 to -10.48	Yes	**	0.0079
♀ Control vs. ♀ S1+N6022	-24.12	-67.00 to 18.75	No	ns	0.5864
♀ S1 vs. ♀ S1+GSNO	50.87	7.996 to 93.74	Yes	*	0.0124
♀ S1 vs. ♀ S1+N6022	80.09	37.22 to 123.0	Yes	****	<0.0001
♀ S1+GSNO vs. ♀ S1+N6022	29.22	-13.65 to 72.09	No	ns	0.3553

Statistical Analysis of figure 6B

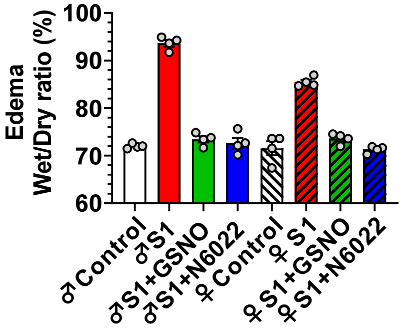

Tukey's multiple comparisons test	Mean Diff.	95.00% CI of diff.	Significant?	Summary	Adjusted P Value
♂ Control vs. ♂ S1	-21.63	-25.37 to -17.90	Yes	****	<0.0001
♂ Control vs. ♂ S1+GSNO	-1.428	-5.162 to 2.307	No	ns	0.9025
♂ Control vs. ♂ S1+N6022	-0.6368	-4.371 to 3.098	No	ns	0.9990
♂ Control vs. ♀ Control	0.4621	-3.272 to 4.197	No	ns	0.9999
♂ Control vs. ♀ S1	-13.48	-17.22 to -9.748	Yes	****	<0.0001
♂ Control vs. ♀ S1+GSNO	-1.568	-5.303 to 2.167	No	ns	0.8527
♂ Control vs. ♀ S1+N6022	0.7246	-3.010 to 4.459	No	ns	0.9978
♂ S1 vs. ♂ S1+GSNO	20.21	16.47 to 23.94	Yes	****	<0.0001
♂ S1 vs. ♂ S1+N6022	21.00	17.26 to 24.73	Yes	****	<0.0001
♂ S1 vs. ♀ Control	22.10	18.36 to 25.83	Yes	****	<0.0001
♂ S1 vs. ♀ S1	8.152	4.417 to 11.89	Yes	****	<0.0001
♂ S1 vs. ♀ S1+GSNO	20.07	16.33 to 23.80	Yes	****	<0.0001
♂ S1 vs. ♀ S1+N6022	22.36	18.62 to 26.09	Yes	****	<0.0001
♂ S1+GSNO vs. ♂ S1+N6022	0.7909	-2.944 to 4.526	No	ns	0.9961
♂ S1+GSNO vs. ♀ Control	1.890	-1.845 to 5.624	No	ns	0.7016
♂ S1+GSNO vs. ♀ S1	-12.06	-15.79 to -8.321	Yes	****	<0.0001
♂ S1+GSNO vs. ♀ S1+GSNO	-0.1404	-3.875 to 3.594	No	ns	>0.9999
♂ S1+GSNO vs. ♀ S1+N6022	2.152	-1.582 to 5.887	No	ns	0.5583
♂ S1+N6022 vs. ♀ Control	1.099	-2.636 to 4.834	No	ns	0.9739
♂ S1+N6022 vs. ♀ S1	-12.85	-16.58 to -9.111	Yes	****	<0.0001
♂ S1+N6022 vs. ♀ S1+GSNO	-0.9313	-4.666 to 2.803	No	ns	0.9897
♂ S1+N6022 vs. ♀ S1+N6022	1.361	-2.373 to 5.096	No	ns	0.9219
♀ Control vs. ♀ S1	-13.94	-17.68 to -10.21	Yes	****	<0.0001
♀ Control vs. ♀ S1+GSNO	-2.030	-5.765 to 1.704	No	ns	0.6257
♀ Control vs. ♀ S1+N6022	0.2625	-3.472 to 3.997	No	ns	>0.9999
♀ S1 vs. ♀ S1+GSNO	11.91	8.180 to 15.65	Yes	****	<0.0001
♀ S1 vs. ♀ S1+N6022	14.21	10.47 to 17.94	Yes	****	<0.0001

Statistical Analysis of figure 6C-ii

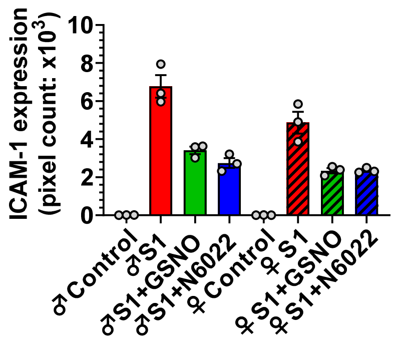

Tukey's multiple comparisons test	Mean Diff.	95.00% CI of diff.	Significant?	Summary	Adjusted P Value
♂ Control vs. ♂ S1	-6.766	-8.330 to -5.203	Yes	****	<0.0001
♂ Control vs. ♂ S1+GSNO	-3.402	-4.966 to -1.839	Yes	****	<0.0001
♂ Control vs. ♂ S1+N6022	-2.739	-4.302 to -1.175	Yes	***	0.0003
♂ Control vs. ♀ Control	0.001333	-1.562 to 1.565	No	ns	>0.9999
♂ Control vs. ♀ S1	-4.859	-6.423 to -3.296	Yes	****	<0.0001
♂ Control vs. ♀ S1+GSNO	-2.331	-3.894 to -0.7677	Yes	**	0.0019
♂ Control vs. ♀ S1+N6022	-2.347	-3.911 to -0.7841	Yes	**	0.0017
♂ S1 vs. ♂ S1+GSNO	3.364	1.801 to 4.927	Yes	****	<0.0001
♂ S1 vs. ♂ S1+N6022	4.028	2.464 to 5.591	Yes	****	<0.0001
♂ S1 vs. ♀ Control	6.768	5.204 to 8.331	Yes	****	<0.0001
♂ S1 vs. ♀ S1	1.907	0.3437 to 3.470	Yes	*	0.0116
♂ S1 vs. ♀ S1+GSNO	4.435	2.872 to 5.999	Yes	****	<0.0001
♂ S1 vs. ♀ S1+N6022	4.419	2.856 to 5.982	Yes	****	<0.0001
♂ S1+GSNO vs. ♂ S1+N6022	0.6637	-0.8996 to 2.227	No	ns	0.8120
♂ S1+GSNO vs. ♀ Control	3.404	1.840 to 4.967	Yes	****	<0.0001
♂ S1+GSNO vs. ♀ S1	-1.457	-3.020 to 0.1063	No	ns	0.0772
♂ S1+GSNO vs. ♀ S1+GSNO	1.071	-0.4919 to 2.635	No	ns	0.3162
♂ S1+GSNO vs. ♀ S1+N6022	1.055	-0.5083 to 2.618	No	ns	0.3328
♂ S1+N6022 vs. ♀ Control	2.740	1.177 to 4.303	Yes	***	0.0003
♂ S1+N6022 vs. ♀ S1	-2.121	-3.684 to -0.5574	Yes	**	0.0046
♂ S1+N6022 vs. ♀ S1+GSNO	0.4077	-1.156 to 1.971	No	ns	0.9814
♂ S1+N6022 vs. ♀ S1+N6022	0.3913	-1.172 to 1.955	No	ns	0.9852
♀ Control vs. ♀ S1	-4.861	-6.424 to -3.297	Yes	****	<0.0001
♀ Control vs. ♀ S1+GSNO	-2.332	-3.896 to -0.7691	Yes	**	0.0019
♀ Control vs. ♀ S1+N6022	-2.349	-3.912 to -0.7854	Yes	**	0.0017
♀ S1 vs. ♀ S1+GSNO	2.528	0.9651 to 4.092	Yes	***	0.0008
♀ S1 vs. ♀ S1+N6022	2.512	0.9487 to 4.075	Yes	***	0.0009
♀ S1+GSNO vs. ♀ S1+N6022	-0.01633	-1.580 to 1.547	No	ns	>0.9999

Statistical Analysis of figure 6C-iii

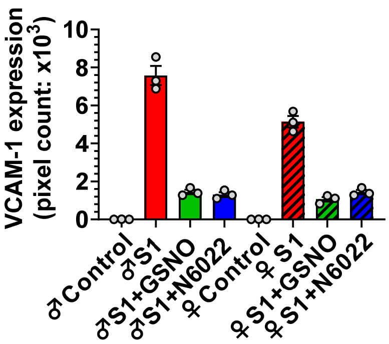

Tukey's multiple comparisons test	Mean Diff.	95.00% CI of diff.	Significant?	Summary	Adjusted P Value
♂ Control vs. ♂ S1	-7.569	-8.673 to -6.464	Yes	****	<0.0001
♂ Control vs. ♂ S1+GSNO	-1.438	-2.542 to -0.3333	Yes	**	0.0067
♂ Control vs. ♂ S1+N6022	-1.300	-2.405 to -0.1960	Yes	*	0.0154
♂ Control vs. ♀ Control	-0.0003333	-1.105 to 1.104	No	ns	>0.9999
♂ Control vs. ♀ S1	-5.136	-6.240 to -4.031	Yes	****	<0.0001
♂ Control vs. ♀ S1+GSNO	-1.054	-2.158 to 0.05065	No	ns	0.0671
♂ Control vs. ♀ S1+N6022	-1.431	-2.536 to -0.3270	Yes	**	0.0069
♂ S1 vs. ♂ S1+GSNO	6.131	5.027 to 7.235	Yes	****	<0.0001
♂ S1 vs. ♂ S1+N6022	6.268	5.164 to 7.373	Yes	****	<0.0001
♂ S1 vs. ♀ Control	7.568	6.464 to 8.673	Yes	****	<0.0001
♂ S1 vs. ♀ S1	2.433	1.329 to 3.537	Yes	****	<0.0001
♂ S1 vs. ♀ S1+GSNO	6.515	5.411 to 7.619	Yes	****	<0.0001
♂ S1 vs. ♀ S1+N6022	6.137	5.033 to 7.242	Yes	****	<0.0001
♂ S1+GSNO vs. ♂ S1+N6022	0.1373	-0.9670 to 1.242	No	ns	0.9998
♂ S1+GSNO vs. ♀ Control	1.437	0.3330 to 2.542	Yes	**	0.0067
♂ S1+GSNO vs. ♀ S1	-3.698	-4.802 to -2.594	Yes	****	<0.0001
♂ S1+GSNO vs. ♀ S1+GSNO	0.3840	-0.7203 to 1.488	No	ns	0.9193
♂ S1+GSNO vs. ♀ S1+N6022	0.006333	-1.098 to 1.111	No	ns	>0.9999
♂ S1+N6022 vs. ♀ Control	1.300	0.1957 to 2.404	Yes	*	0.0154
♂ S1+N6022 vs. ♀ S1	-3.835	-4.940 to -2.731	Yes	****	<0.0001
♂ S1+N6022 vs. ♀ S1+GSNO	0.2467	-0.8577 to 1.351	No	ns	0.9923
♂ S1+N6022 vs. ♀ S1+N6022	-0.1310	-1.235 to 0.9733	No	ns	0.9999
♀ Control vs. ♀ S1	-5.135	-6.240 to -4.031	Yes	****	<0.0001
♀ Control vs. ♀ S1+GSNO	-1.053	-2.158 to 0.05098	No	ns	0.0673
♀ Control vs. ♀ S1+N6022	-1.431	-2.535 to -0.3267	Yes	**	0.0069
♀ S1 vs. ♀ S1+GSNO	4.082	2.978 to 5.186	Yes	****	<0.0001
♀ S1 vs. ♀ S1+N6022	3.704	2.600 to 4.809	Yes	****	<0.0001
♀ S1+GSNO vs. ♀ S1+N6022	-0.3777	-1.482 to 0.7267	No	ns	0.9253

Statistical Analysis of figure 7A-i

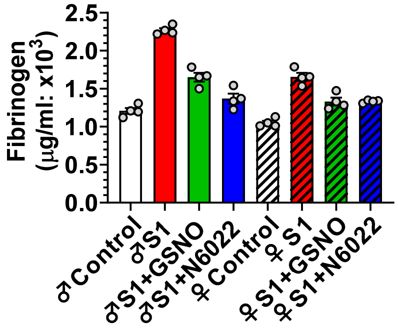

Tukey's multiple comparisons test	Mean Diff.	95.00% CI of diff.	Significant?	Summary	Adjusted P Value
♂ Control vs. ♂ S1	-1.059	-1.270 to -0.8489	Yes	****	<0.0001
♂ Control vs. ♂ S1+GSNO	-0.4427	-0.6530 to -0.2323	Yes	****	<0.0001
♂ Control vs. ♂ S1+N6022	-0.1605	-0.3709 to 0.04986	No	ns	0.2316
♂ Control vs. ♀ Control	0.1582	-0.05221 to 0.3685	No	ns	0.2466
♂ Control vs. ♀ S1	-0.4476	-0.6579 to -0.2372	Yes	****	<0.0001
♂ Control vs. ♀ S1+GSNO	-0.1221	-0.3324 to 0.08830	No	ns	0.5502
♂ Control vs. ♀ S1+N6022	-0.1240	-0.3343 to 0.08642	No	ns	0.5319
♂ S1 vs. ♂ S1+GSNO	0.6166	0.4062 to 0.8270	Yes	****	<0.0001
♂ S1 vs. ♂ S1+N6022	0.8987	0.6884 to 1.109	Yes	****	<0.0001
♂ S1 vs. ♀ Control	1.217	1.007 to 1.428	Yes	****	<0.0001
♂ S1 vs. ♀ S1	0.6117	0.4013 to 0.8220	Yes	****	<0.0001
♂ S1 vs. ♀ S1+GSNO	0.9372	0.7268 to 1.148	Yes	****	<0.0001
♂ S1 vs. ♀ S1+N6022	0.9353	0.7249 to 1.146	Yes	****	<0.0001
♂ S1+GSNO vs. ♂ S1+N6022	0.2821	0.07177 to 0.4925	Yes	**	0.0036
♂ S1+GSNO vs. ♀ Control	0.6008	0.3904 to 0.8112	Yes	****	<0.0001
♂ S1+GSNO vs. ♀ S1	-0.004919	-0.2153 to 0.2055	No	ns	>0.9999
♂ S1+GSNO vs. ♀ S1+GSNO	0.3206	0.1102 to 0.5310	Yes	***	0.0008
♂ S1+GSNO vs. ♀ S1+N6022	0.3187	0.1083 to 0.5291	Yes	***	0.0009
♂ S1+N6022 vs. ♀ Control	0.3187	0.1083 to 0.5290	Yes	***	0.0009
♂ S1+N6022 vs. ♀ S1	-0.2871	-0.4974 to -0.07668	Yes	**	0.0030
♂ S1+N6022 vs. ♀ S1+GSNO	0.03844	-0.1719 to 0.2488	No	ns	0.9985
♂ S1+N6022 vs. ♀ S1+N6022	0.03656	-0.1738 to 0.2469	No	ns	0.9989
♀ Control vs. ♀ S1	-0.6057	-0.8161 to -0.3954	Yes	****	<0.0001
♀ Control vs. ♀ S1+GSNO	-0.2802	-0.4906 to -0.06986	Yes	**	0.0039
♀ Control vs. ♀ S1+N6022	-0.2821	-0.4925 to -0.07174	Yes	**	0.0036
♀ S1 vs. ♀ S1+GSNO	0.3255	0.1151 to 0.5359	Yes	***	0.0007
♀ S1 vs. ♀ S1+N6022	0.3236	0.1132 to 0.5340	Yes	***	0.0007
♀ S1+GSNO vs. ♀ S1+N6022	-0.001880	-0.2123 to 0.2085	No	ns	>0.9999

Statistical Analysis of figure 7A-ii

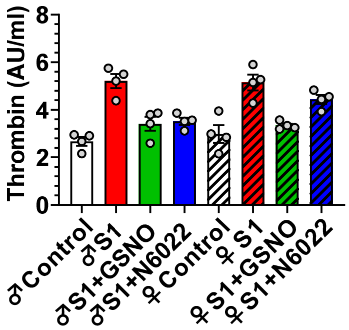

Tukey's multiple comparisons test	Mean Diff.	95.00% CI of diff.	Significant?	Summary	Adjusted P Value
♂ Control vs. ♂ S1	-2.537	-3.733 to -1.340	Yes	****	<0.0001
♂ Control vs. ♂ S1+GSNO	-0.7460	-1.942 to 0.4502	No	ns	0.4628
♂ Control vs. ♂ S1+N6022	-0.8427	-2.039 to 0.3536	No	ns	0.3172
♂ Control vs. ♀ Control	-0.3083	-1.505 to 0.8879	No	ns	0.9876
♂ Control vs. ♀ S1	-2.480	-3.676 to -1.284	Yes	****	<0.0001
♂ Control vs. ♀ S1+GSNO	-0.6620	-1.858 to 0.5342	No	ns	0.6056
♂ Control vs. ♀ S1+N6022	-1.764	-2.960 to -0.5674	Yes	**	0.0012
♂ S1 vs. ♂ S1+GSNO	1.791	0.5944 to 2.987	Yes	**	0.0010
♂ S1 vs. ♂ S1+N6022	1.694	0.4978 to 2.890	Yes	**	0.0020
♂ S1 vs. ♀ Control	2.228	1.032 to 3.425	Yes	****	<0.0001
♂ S1 vs. ♀ S1	0.05667	-1.140 to 1.253	No	ns	>0.9999
♂ S1 vs. ♀ S1+GSNO	1.875	0.6784 to 3.071	Yes	***	0.0006
♂ S1 vs. ♀ S1+N6022	0.7730	-0.4232 to 1.969	No	ns	0.4194
♂ S1+GSNO vs. ♂ S1+N6022	-0.09667	-1.293 to 1.100	No	ns	>0.9999
♂ S1+GSNO vs. ♀ Control	0.4377	-0.7586 to 1.634	No	ns	0.9205
♂ S1+GSNO vs. ♀ S1	-1.734	-2.930 to -0.5378	Yes	**	0.0015
♂ S1+GSNO vs. ♀ S1+GSNO	0.08400	-1.112 to 1.280	No	ns	>0.9999
♂ S1+GSNO vs. ♀ S1+N6022	-1.018	-2.214 to 0.1786	No	ns	0.1371
♂ S1+N6022 vs. ♀ Control	0.5343	-0.6619 to 1.731	No	ns	0.8106
♂ S1+N6022 vs. ♀ S1	-1.637	-2.834 to -0.4411	Yes	**	0.0029
♂ S1+N6022 vs. ♀ S1+GSNO	0.1807	-1.016 to 1.377	No	ns	0.9996
♂ S1+N6022 vs. ♀ S1+N6022	-0.9210	-2.117 to 0.2752	No	ns	0.2227
♀ Control vs. ♀ S1	-2.172	-3.368 to -0.9754	Yes	****	<0.0001
♀ Control vs. ♀ S1+GSNO	-0.3537	-1.550 to 0.8426	No	ns	0.9732
♀ Control vs. ♀ S1+N6022	-1.455	-2.652 to -0.2591	Yes	**	0.0098
♀ S1 vs. ♀ S1+GSNO	1.818	0.6218 to 3.014	Yes	***	0.0009
♀ S1 vs. ♀ S1+N6022	0.7163	-0.4799 to 1.913	No	ns	0.5123
♀ S1+GSNO vs. ♀ S1+N6022	-1.102	-2.298 to 0.09457	No	ns	0.0866

Statistical Analysis of figure 7A-iii

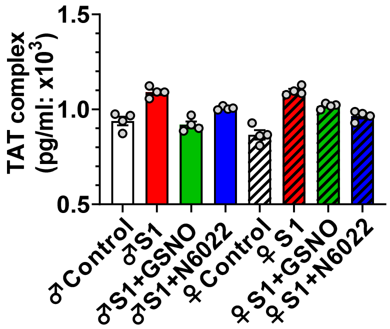

Tukey's multiple comparisons test	Mean Diff.	95.00% CI of diff.	Significant?	Summary	Adjusted P Value
♂ Control vs. ♂ S1	-0.1518	-0.2261 to -0.07748	Yes	****	<0.0001
♂ Control vs. ♂ S1+GSNO	0.02003	-0.05426 to 0.09431	No	ns	0.9839
♂ Control vs. ♂ S1+N6022	-0.06753	-0.1418 to 0.006754	No	ns	0.0938
♂ Control vs. ♀ Control	0.07252	-0.001769 to 0.1468	No	ns	0.0592
♂ Control vs. ♀ S1	-0.1578	-0.2320 to -0.08347	Yes	****	<0.0001
♂ Control vs. ♀ S1+GSNO	-0.07889	-0.1532 to -0.004605	Yes	*	0.0318
♂ Control vs. ♀ S1+N6022	-0.02653	-0.1008 to 0.04775	No	ns	0.9291
♂ S1 vs. ♂ S1+GSNO	0.1718	0.09751 to 0.2461	Yes	****	<0.0001
♂ S1 vs. ♂ S1+N6022	0.08424	0.009953 to 0.1585	Yes	*	0.0185
♂ S1 vs. ♀ Control	0.2243	0.1500 to 0.2986	Yes	****	<0.0001
♂ S1 vs. ♀ S1	-0.005985	-0.08027 to 0.06830	No	ns	>0.9999
♂ S1 vs. ♀ S1+GSNO	0.07288	-0.001406 to 0.1472	No	ns	0.0572
♂ S1 vs. ♀ S1+N6022	0.1252	0.05095 to 0.1995	Yes	***	0.0002
♂ S1+GSNO vs. ♂ S1+N6022	-0.08756	-0.1618 to -0.01327	Yes	*	0.0131
♂ S1+GSNO vs. ♀ Control	0.05249	-0.02180 to 0.1268	No	ns	0.3137
♂ S1+GSNO vs. ♀ S1	-0.1778	-0.2521 to -0.1035	Yes	****	<0.0001
♂ S1+GSNO vs. ♀ S1+GSNO	-0.09892	-0.1732 to -0.02463	Yes	**	0.0039
♂ S1+GSNO vs. ♀ S1+N6022	-0.04656	-0.1208 to 0.02772	No	ns	0.4566
♂ S1+N6022 vs. ♀ Control	0.1400	0.06576 to 0.2143	Yes	****	<0.0001
♂ S1+N6022 vs. ♀ S1	-0.09022	-0.1645 to -0.01594	Yes	**	0.0099
♂ S1+N6022 vs. ♀ S1+GSNO	-0.01136	-0.08564 to 0.06293	No	ns	0.9995
♂ S1+N6022 vs. ♀ S1+N6022	0.04100	-0.03329 to 0.1153	No	ns	0.6087
♀ Control vs. ♀ S1	-0.2303	-0.3046 to -0.1560	Yes	****	<0.0001
♀ Control vs. ♀ S1+GSNO	-0.1514	-0.2257 to -0.07712	Yes	****	<0.0001
♀ Control vs. ♀ S1+N6022	-0.09905	-0.1733 to -0.02477	Yes	**	0.0039
♀ S1 vs. ♀ S1+GSNO	0.07886	0.004579 to 0.1531	Yes	*	0.0319
♀ S1 vs. ♀ S1+N6022	0.1312	0.05693 to 0.2055	Yes	***	0.0001
♀ S1+GSNO vs. ♀ S1+N6022	0.05236	-0.02193 to 0.1266	No	ns	0.3166

Statistical Analysis of figure 7A-iv

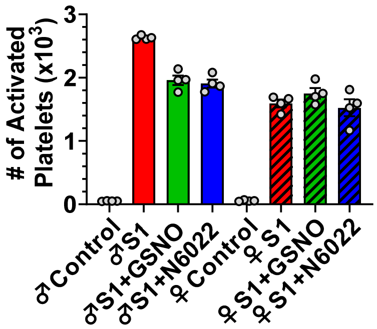

Tukey's multiple comparisons test	Mean Diff.	95.00% CI of diff.	Significant?	Summary	Adjusted P Value
♂ Control vs. ♂ S1	-2.579	-2.905 to -2.254	Yes	****	<0.0001
♂ Control vs. ♂ S1+GSNO	-1.908	-2.233 to -1.582	Yes	****	<0.0001
♂ Control vs. ♂ S1+N6022	-1.857	-2.182 to -1.532	Yes	****	<0.0001
♂ Control vs. ♀ Control	-0.005250	-0.3307 to 0.3202	No	ns	>0.9999
♂ Control vs. ♀ S1	-1.542	-1.868 to -1.217	Yes	****	<0.0001
♂ Control vs. ♀ S1+GSNO	-1.702	-2.027 to -1.376	Yes	****	<0.0001
♂ Control vs. ♀ S1+N6022	-1.473	-1.799 to -1.148	Yes	****	<0.0001
♂ S1 vs. ♂ S1+GSNO	0.6718	0.3463 to 0.9972	Yes	****	<0.0001
♂ S1 vs. ♂ S1+N6022	0.7223	0.3968 to 1.048	Yes	****	<0.0001
♂ S1 vs. ♀ Control	2.574	2.249 to 2.899	Yes	****	<0.0001
♂ S1 vs. ♀ S1	1.037	0.7116 to 1.362	Yes	****	<0.0001
♂ S1 vs. ♀ S1+GSNO	0.8778	0.5523 to 1.203	Yes	****	<0.0001
♂ S1 vs. ♀ S1+N6022	1.106	0.7806 to 1.431	Yes	****	<0.0001
♂ S1+GSNO vs. ♂ S1+N6022	0.05050	-0.2749 to 0.3759	No	ns	0.9995
♂ S1+GSNO vs. ♀ Control	1.902	1.577 to 2.228	Yes	****	<0.0001
♂ S1+GSNO vs. ♀ S1	0.3653	0.03982 to 0.6907	Yes	*	0.0202
♂ S1+GSNO vs. ♀ S1+GSNO	0.2060	-0.1194 to 0.5314	No	ns	0.4446
♂ S1+GSNO vs. ♀ S1+N6022	0.4343	0.1088 to 0.7597	Yes	**	0.0038
♂ S1+N6022 vs. ♀ Control	1.852	1.526 to 2.177	Yes	****	<0.0001
♂ S1+N6022 vs. ♀ S1	0.3148	-0.01068 to 0.6402	No	ns	0.0631
♂ S1+N6022 vs. ♀ S1+GSNO	0.1555	-0.1699 to 0.4809	No	ns	0.7556
♂ S1+N6022 vs. ♀ S1+N6022	0.3838	0.05832 to 0.7092	Yes	*	0.0131
♀ Control vs. ♀ S1	-1.537	-1.862 to -1.212	Yes	****	<0.0001
♀ Control vs. ♀ S1+GSNO	-1.696	-2.022 to -1.371	Yes	****	<0.0001
♀ Control vs. ♀ S1+N6022	-1.468	-1.793 to -1.143	Yes	****	<0.0001
♀ S1 vs. ♀ S1+GSNO	-0.1593	-0.4847 to 0.1662	No	ns	0.7340
♀ S1 vs. ♀ S1+N6022	0.06900	-0.2564 to 0.3944	No	ns	0.9961
♀ S1+GSNO vs. ♀ S1+N6022	0.2283	-0.09718 to 0.5537	No	ns	0.3221

Statistical Analysis of figure 7A-v

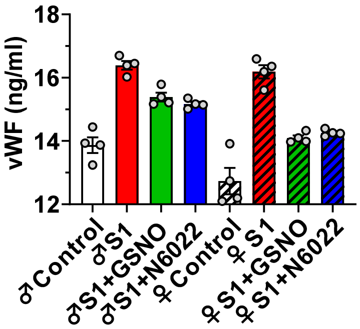

Tukey's multiple comparisons test	Mean Diff.	95.00% CI of diff.	Significant?	Summary	Adjusted P Value
♂ Control vs. ♂ S1	-2.519	-3.472 to -1.565	Yes	****	<0.0001
♂ Control vs. ♂ S1+GSNO	-1.518	-2.472 to -0.5640	Yes	***	0.0005
♂ Control vs. ♂ S1+N6022	-1.299	-2.253 to -0.3455	Yes	**	0.0031
♂ Control vs. ♀ Control	1.135	0.1814 to 2.089	Yes	*	0.0120
♂ Control vs. ♀ S1	-2.315	-3.269 to -1.361	Yes	****	<0.0001
♂ Control vs. ♀ S1+GSNO	-0.2257	-1.180 to 0.7282	No	ns	0.9925
♂ Control vs. ♀ S1+N6022	-0.3891	-1.343 to 0.5648	No	ns	0.8698
♂ S1 vs. ♂ S1+GSNO	1.001	0.04687 to 1.955	Yes	*	0.0350
♂ S1 vs. ♂ S1+N6022	1.219	0.2654 to 2.173	Yes	**	0.0060
♂ S1 vs. ♀ Control	3.654	2.700 to 4.608	Yes	****	<0.0001
♂ S1 vs. ♀ S1	0.2033	-0.7505 to 1.157	No	ns	0.9960
♂ S1 vs. ♀ S1+GSNO	2.293	1.339 to 3.247	Yes	****	<0.0001
♂ S1 vs. ♀ S1+N6022	2.130	1.176 to 3.083	Yes	****	<0.0001
♂ S1+GSNO vs. ♂ S1+N6022	0.2186	-0.7353 to 1.172	No	ns	0.9938
♂ S1+GSNO vs. ♀ Control	2.653	1.699 to 3.607	Yes	****	<0.0001
♂ S1+GSNO vs. ♀ S1	-0.7974	-1.751 to 0.1564	No	ns	0.1503
♂ S1+GSNO vs. ♀ S1+GSNO	1.292	0.3384 to 2.246	Yes	**	0.0033
♂ S1+GSNO vs. ♀ S1+N6022	1.129	0.1750 to 2.083	Yes	*	0.0127
♂ S1+N6022 vs. ♀ Control	2.435	1.481 to 3.388	Yes	****	<0.0001
♂ S1+N6022 vs. ♀ S1	-1.016	-1.970 to -0.06215	Yes	*	0.0311
♂ S1+N6022 vs. ♀ S1+GSNO	1.074	0.1198 to 2.027	Yes	*	0.0198
♂ S1+N6022 vs. ♀ S1+N6022	0.9102	-0.04362 to 1.864	No	ns	0.0690
♀ Control vs. ♀ S1	-3.450	-4.404 to -2.497	Yes	****	<0.0001
♀ Control vs. ♀ S1+GSNO	-1.361	-2.315 to -0.4071	Yes	**	0.0018
♀ Control vs. ♀ S1+N6022	-1.524	-2.478 to -0.5704	Yes	***	0.0005
♀ S1 vs. ♀ S1+GSNO	2.090	1.136 to 3.043	Yes	****	<0.0001
♀ S1 vs. ♀ S1+N6022	1.926	0.9724 to 2.880	Yes	****	<0.0001
♀ S1+GSNO vs. ♀ S1+N6022	-0.1634	-1.117 to 0.7904	No	ns	0.9990

Statistical Analysis of figure 7A-vi

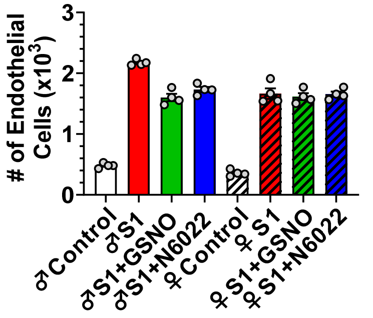

Tukey's multiple comparisons test	Mean Diff.	95.00% CI of diff.	Significant?	Summary	Adjusted P Value
♂ Control vs. ♂ S1	-1.694	-1.926 to -1.462	Yes	****	<0.0001
♂ Control vs. ♂ S1+GSNO	-1.121	-1.353 to -0.8885	Yes	****	<0.0001
♂ Control vs. ♂ S1+N6022	-1.251	-1.483 to -1.019	Yes	****	<0.0001
♂ Control vs. ♀ Control	0.1240	-0.1083 to 0.3563	No	ns	0.6458
♂ Control vs. ♀ S1	-1.185	-1.417 to -0.9522	Yes	****	<0.0001
♂ Control vs. ♀ S1+GSNO	-1.135	-1.367 to -0.9022	Yes	****	<0.0001
♂ Control vs. ♀ S1+N6022	-1.176	-1.408 to -0.9435	Yes	****	<0.0001
♂ S1 vs. ♂ S1+GSNO	0.5733	0.3410 to 0.8055	Yes	****	<0.0001
♂ S1 vs. ♂ S1+N6022	0.4430	0.2107 to 0.6753	Yes	****	<0.0001
♂ S1 vs. ♀ Control	1.818	1.586 to 2.050	Yes	****	<0.0001
♂ S1 vs. ♀ S1	0.5095	0.2772 to 0.7418	Yes	****	<0.0001
♂ S1 vs. ♀ S1+GSNO	0.5595	0.3272 to 0.7918	Yes	****	<0.0001
♂ S1 vs. ♀ S1+N6022	0.5183	0.2860 to 0.7505	Yes	****	<0.0001
♂ S1+GSNO vs. ♂ S1+N6022	-0.1303	-0.3625 to 0.1020	No	ns	0.5905
♂ S1+GSNO vs. ♀ Control	1.245	1.012 to 1.477	Yes	****	<0.0001
♂ S1+GSNO vs. ♀ S1	-0.06375	-0.2960 to 0.1685	No	ns	0.9822
♂ S1+GSNO vs. ♀ S1+GSNO	-0.01375	-0.2460 to 0.2185	No	ns	>0.9999
♂ S1+GSNO vs. ♀ S1+N6022	-0.05500	-0.2873 to 0.1773	No	ns	0.9924
♂ S1+N6022 vs. ♀ Control	1.375	1.143 to 1.607	Yes	****	<0.0001
♂ S1+N6022 vs. ♀ S1	0.06650	-0.1658 to 0.2988	No	ns	0.9775
♂ S1+N6022 vs. ♀ S1+GSNO	0.1165	-0.1158 to 0.3488	No	ns	0.7105
♂ S1+N6022 vs. ♀ S1+N6022	0.07525	-0.1570 to 0.3075	No	ns	0.9565
♀ Control vs. ♀ S1	-1.309	-1.541 to -1.076	Yes	****	<0.0001
♀ Control vs. ♀ S1+GSNO	-1.259	-1.491 to -1.026	Yes	****	<0.0001
♀ Control vs. ♀ S1+N6022	-1.300	-1.532 to -1.067	Yes	****	<0.0001
♀ S1 vs. ♀ S1+GSNO	0.05000	-0.1823 to 0.2823	No	ns	0.9957
♀ S1 vs. ♀ S1+N6022	0.008750	-0.2235 to 0.2410	No	ns	>0.9999
♀ S1+GSNO vs. ♀ S1+N6022	-0.04125	-0.2735 to 0.1910	No	ns	0.9987

Statistical Analysis of figure 7B

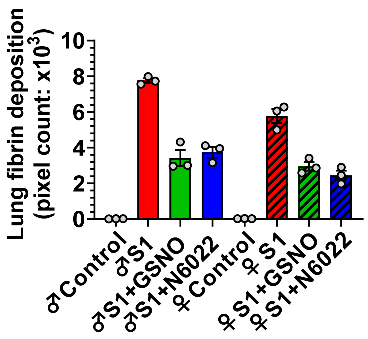

Tukey's multiple comparisons test	Mean Diff.	95.00% CI of diff.	Significant?	Summary	Adjusted P Value
♂ Control vs. ♂ S1	-7.745	-9.073 to -6.417	Yes	****	<0.0001
♂ Control vs. ♂ S1+GSNO	-3.415	-4.743 to -2.086	Yes	****	<0.0001
♂ Control vs. ♂ S1+N6022	-3.724	-5.052 to -2.396	Yes	****	<0.0001
♂ Control vs. ♀ Control	-0.004667	-1.333 to 1.324	No	ns	>0.9999
♂ Control vs. ♀ S1	-5.760	-7.088 to -4.431	Yes	****	<0.0001
♂ Control vs. ♀ S1+GSNO	-2.935	-4.263 to -1.606	Yes	****	<0.0001
♂ Control vs. ♀ S1+N6022	-2.424	-3.752 to -1.096	Yes	***	0.0002
♂ S1 vs. ♂ S1+GSNO	4.330	3.002 to 5.659	Yes	****	<0.0001
♂ S1 vs. ♂ S1+N6022	4.021	2.693 to 5.349	Yes	****	<0.0001
♂ S1 vs. ♀ Control	7.740	6.412 to 9.069	Yes	****	<0.0001
♂ S1 vs. ♀ S1	1.985	0.6571 to 3.314	Yes	**	0.0018
♂ S1 vs. ♀ S1+GSNO	4.810	3.482 to 6.139	Yes	****	<0.0001
♂ S1 vs. ♀ S1+N6022	5.321	3.993 to 6.649	Yes	****	<0.0001
♂ S1+GSNO vs. ♂ S1+N6022	-0.3093	-1.638 to 1.019	No	ns	0.9902
♂ S1+GSNO vs. ♀ Control	3.410	2.082 to 4.738	Yes	****	<0.0001
♂ S1+GSNO vs. ♀ S1	-2.345	-3.673 to -1.017	Yes	***	0.0003
♂ S1+GSNO vs. ♀ S1+GSNO	0.4800	-0.8482 to 1.808	No	ns	0.9038
♂ S1+GSNO vs. ♀ S1+N6022	0.9907	-0.3375 to 2.319	No	ns	0.2313
♂ S1+N6022 vs. ♀ Control	3.719	2.391 to 5.048	Yes	****	<0.0001
♂ S1+N6022 vs. ♀ S1	-2.036	-3.364 to -0.7075	Yes	**	0.0014
♂ S1+N6022 vs. ♀ S1+GSNO	0.7893	-0.5389 to 2.118	No	ns	0.4778
♂ S1+N6022 vs. ♀ S1+N6022	1.300	-0.02821 to 2.628	No	ns	0.0573
♀ Control vs. ♀ S1	-5.755	-7.083 to -4.427	Yes	****	<0.0001
♀ Control vs. ♀ S1+GSNO	-2.930	-4.258 to -1.602	Yes	****	<0.0001
♀ Control vs. ♀ S1+N6022	-2.419	-3.748 to -1.091	Yes	***	0.0002
♀ S1 vs. ♀ S1+GSNO	2.825	1.497 to 4.153	Yes	****	<0.0001
♀ S1 vs. ♀ S1+N6022	3.336	2.007 to 4.664	Yes	****	<0.0001
♀ S1+GSNO vs. ♀ S1+N6022	0.5107	-0.8175 to 1.839	No	ns	0.8740
